# Fraxin Attenuates Rheumatoid Arthritis by Regulating Macrophage Polarization and Inhibiting Fibroblast-like Synoviocyte Proliferation

**DOI:** 10.3390/ijms27072946

**Published:** 2026-03-24

**Authors:** Anjing Xu, Bao Hou, Shijie Zhang, Xiaoyue Ma, Yuanyuan Wen, Xuexue Zhu, Weiwei Cai, Jing Chen, Ma Mi, Tsedien Nhamdrie, Liying Qiu, Haijian Sun, Minhui Hua

**Affiliations:** 1 Department of Rheumatology, Jiangnan University Medical Center (Wuxi No. 2 People’s Hospital), Wuxi School of Medicine, Jiangnan University, Wuxi 214002, China; 6232819002@stu.jiangnan.edu.cn (A.X.);; 2 MOE Medical Basic Research Innovation Center for Gut Microbiota and Chronic Diseases, School of Medicine, Jiangnan University, Wuxi 214122, China; 3 Department of Basic Medicine, Wuxi School of Medicine, Jiangnan University, Wuxi 214122, China; 4 Department of Basic Medicine, Tibet University of Medicine, Lhasa 850000, China; 5 State Key Laboratory of Natural Medicines, China Pharmaceutical University, No. 24 Tongjia Lane, Nanjing 210009, China

**Keywords:** Fraxin, HSPA8, rheumatoid arthritis, fibroblast-like synovial cell, network pharmacology

## Abstract

Wuweiganlu (WGL) is a traditional formulation widely applied in the treatment of rheumatoid arthritis (RA), yet the identity of its bioactive constituents remains inadequately defined. In this study, ultra-performance liquid chromatography coupled with quadrupole time-of-flight mass spectrometry (UPLC-Q-TOF-MS) and untargeted serum metabolomics were employed to characterize the active components of WGL. Fraxin was identified as a principal compound from WGL. To investigate its therapeutic mechanism in RA, a series of in silico and experimental approaches were conducted. Network pharmacology analysis and RNA sequencing identified heat shock protein family member 8 (HSPA8) as a potential molecular target of Fraxin, which was further validated by molecular docking studies. Gene Ontology (GO) and Kyoto Encyclopedia of Genes and Genomes (KEGG) enrichment analyses indicated that Fraxin exerts its effects primarily by modulating cell apoptosis through the PI3K signaling pathway. In vitro experiments demonstrated that Fraxin significantly reduced inflammatory responses and downregulated HSPA8 expression in lipopolysaccharide (LPS)-stimulated fibroblast-like synoviocytes (FLs) and macrophages. In vivo, Fraxin administration markedly reduced paw swelling, alleviated bone deformities, and improved bone volume fraction (BV/TV) in male IL1RA-deficient mice exhibiting spontaneous arthritis. Histological analysis confirmed that Fraxin attenuated joint inflammation by modulating the inflammatory microenvironment. Additionally, Fraxin inhibited synovial hyperplasia by regulating mitochondrial membrane potential collapse in FLs. Functional assays revealed that this regulation occurred via the inhibition of HSPA8/PI3K/AKT signaling axis, thereby suppressing aberrant FLS proliferation and contributing to the attenuation of RA progression.

## 1. Introduction

Rheumatoid arthritis (RA) is a chronic, systemic autoimmune disease marked by persistent synovial inflammation, reduced bone mineral density, and progressive bone erosion, affecting approximately 1% of the global population [1,2]. Patients with RA experience sustained joint pain, contributing not only to physical disability and psychological stress [3,4]. While the exact etiology of RA remains incompletely understood, genetic predisposition [5], obesity [6], and smoking [7] are believed to be contributing factors in RA. Current treatments mainly rely on disease-modifying anti-rheumatic drugs (DMARDs) and glucocorticoids (GCs). More recently, biologic agents targeting inflammatory cytokines such as TNF-α and IL-6 have shown remarkable clinical efficacy [8]. However, the high cost of biologics and the adverse effects associated with long-term corticosteroid use, including cardiovascular disease, diabetes, and osteoporosis, highlight the need for safer, more effective, and more affordable therapeutic alternatives.

Traditional Chinese Medicine (TCM), with its holistic approach, has shown promise as a complementary treatment for RA [9,10]. For instance, Chinese Miao Medicine Sidaxue has shown efficacy in collagen-induced arthritis (CIA) models by modulating the VEGF/PI3K/AKT, TNF-α, and IL/NF-κB signaling pathways [11]. Similarly, Wutou Decoction alleviates RA symptoms by inhibiting joint angiogenesis through regulation of the PI3K/AKT/mTOR/HIF-1α signaling cascade [12]. Wuweiganlu (WGL), a Tibetan medicinal compound, is recognized for its therapeutic effect in RA [13]. Our research group demonstrated that aqueous extracts of WGL mitigated experimental arthritis by promoting macrophage polarization toward the M2 phenotype [14]. WGL ethyl acetate extract blocked the development of experimental arthritis by inhibiting NLRP3 activation [15].

Fibroblast-like synoviocytes (FLs), the predominant cell population in the synovial membrane, exhibit enhanced proliferation in response to inflammatory stimuli in RA [16,17]. The pathological features of RA include abnormal proliferation of synovial cells, infiltration of inflammatory cells, formation of rheumatoid pannus, erosion of cartilage and bone, and then lead to joint deformity and bone mineral density loss [18]. Therefore, targeting the synovial inflammatory microenvironment and inhibiting abnormal FLS proliferation may represent an effective therapeutic strategy for RA [19,20].

Fraxin, a coumarin glycoside derived from *Acer tegmentosum* and *Fraxinus ornus*, has demonstrated antioxidant, anti-inflammatory, and anti-metastatic properties. For example, Fraxin mitigates hepatotoxicity by alleviating oxidative stress [21]. Fraxin protects mice against endotoxic shock by inhibiting lipopolysaccharide (LPS)-induced inflammatory cytokine production [22]. Fraxin inhibits the NF-κB and NLRP3 signaling pathways to reduce LPS-induced acute lung injury in mice [23]. Despite these pharmacological effects, the role of Fraxin in RA has not yet been explored.

In the present study, UPLC-Q-TOF-MS and untargeted metabolomics were used to identify bioactive compounds in WGL that may be relevant to RA treatment. Potential targets and signaling pathways of Fraxin were investigated through network pharmacology analysis and RNA sequencing. Its biological effects were then evaluated in cultured FLs and in male IL1RA-deficient mice with spontaneous arthritis. To further clarify the underlying mechanism, both gain- and loss-of-function experiments were performed. These findings provide experimental evidence supporting the potential of Fraxin as a therapeutic candidate for RA.

## 2. Results

### 2.1. Screening of Active Components and Core Targets of WGL in RA

**Figure 1:**
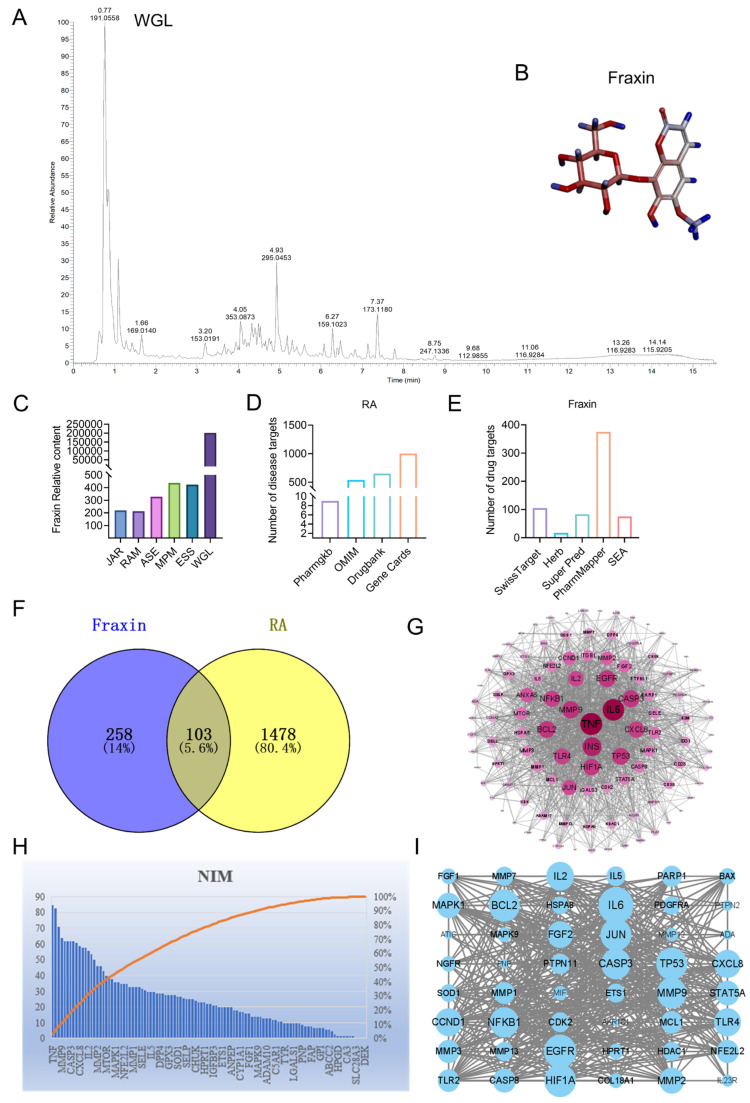
Identification of active components and core targets of WGL in RA. (**A**) WGL total ion map. (**B**) The 3D chemical structure of Fraxin. (**C**) The content of Fraxin in individual herbs and WGL compounds. (**D**) Statistical charts of target points in Fraxin databases. (**E**) Statistical diagram of target points in RA databases. (**F**) Venn diagram of the intersection between Fraxin target library and RA target library. (**G**) PPI network diagram of the targets of Fraxin in the treatment of RA. (**H**) Excerpts of important nodal proteins. (**I**) Important node diagram of the target gene network (Nim).

To identify bioactive compounds within WGL that may be relevant to RA treatment, all five constituent herbs, *Rhododendron anthopogonoides Maxim* (RAM), *Juniperus angosturana R.P. Adams* (JAR), *Ephedra sinica Stapf* (ESS), *Myricaria platyphylla Maxim* (MPM), and *Artemisia sieversiana Ehrh* (ASE), as well as the WGL composite extract, were analyzed using UPLC-Q-TOF-MS (Figure 1A). A total of 28 compounds were detected at concentrations over 50-fold higher in the WGL mixture compared to the individual herbs [15]. Among these, Fraxin, a coumarin with a molecular weight of 370.308 (Figure 1B), possessed anti-inflammatory effects [22,23,24]. In addition, the abundance of Fraxin was significantly higher in WGL than in each individual herbal component (Figure 1C).

To further investigate potential molecular targets of Fraxin in RA, a combined analysis of drug and disease targets was performed. Using the keywords “RA” and “Fraxin,” candidate targets were retrieved from multiple databases, and statistical distributions were visualized (Figure 1D,E). After merging the datasets and removing duplicate entries, 1581 RA-related and 362 Fraxin-related targets were identified, with 103 overlapping targets between the two groups (Figure 1F). These intersecting targets were considered potential mediators of Fraxin’s effect in RA. A protein–protein interaction (PPI) network was subsequently constructed using the STRING database, with node size reflecting the relative centrality of each protein (Figure 1G). To assess node significance, NIM and MCODE correlation algorithms were applied (Figure 1H,I). Integration of results from all three approaches, followed by removal of duplicates, indicated that TNF-α, NF-κB, and HSPA8 may serve as core therapeutic targets of Fraxin in RA.

### 2.2. Transcriptome Sequencing Identified Hspa8 and the PI3K-Akt Signaling Pathway as Key Mechanistic Elements

**Figure 2:**
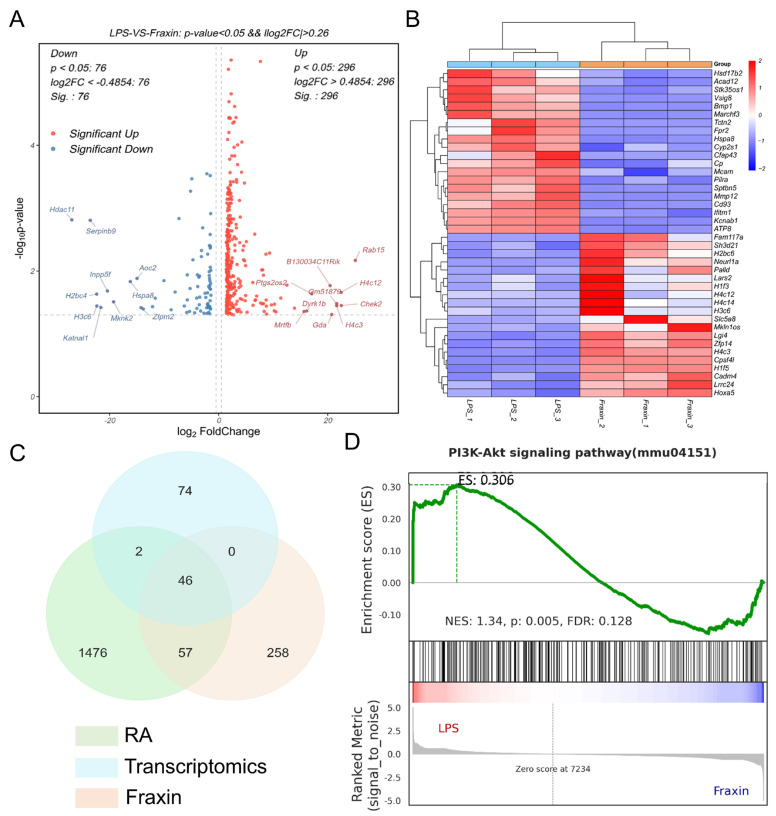
Transcriptome sequencing revealed that Fraxin might regulate the PI3K/Akt signaling pathway. (**A**) The differentially expressed genes between the LPS group and the LPS + Fraxin group in FLs. (**B**) Heatmap of differentially expressed genes between the LPS group and the LPS + Fraxin group in FLs. (**C**) Venn diagram of the intersection between Fraxin target library, RA target library, and the differentially expressed gene library from transcriptomics sequencing. (**D**) Gene set enrichment analysis of the PI3K/Akt signaling pathway.

To investigate the molecular mechanisms by which Fraxin influences RA pathology, transcriptome sequencing was performed in FLs subjected to LPS stimulation with or without Fraxin treatment. Differential gene expression analysis revealed that, following Fraxin intervention, 296 genes were significantly upregulated and 76 were downregulated compared to the LPS-stimulated group. A volcano plot and heatmap illustrated these differentially expressed genes (DEGs), with *Hspa8* exhibiting notable expression changes (Figure 2A,B). To further identify key candidate genes, a Venn diagram analysis was performed by integrating the Fraxin target set, the RA-related gene set, and the DEG dataset derived from transcriptome sequencing. This analysis identified 46 shared genes, including *Hspa8* (Figure 2C), suggesting that Hspa8 may be an important mediator of the effects of Fraxin.

To further interpret the biological relevance of these findings, gene set enrichment analysis (GSEA) was performed using the KEGG database. Results indicated that the inhibitory effect of Fraxin on abnormal FLS proliferation was significantly associated with the PI3K-Akt signaling pathway. The maximum enrichment score (ES) was 0.306, with a normalized enrichment score (NES) of 1.34, a *p*-value of 0.005, and a false discovery rate (FDR) of 0.128. The ranking metric, based on the signal-to-noise ratio, positioned the zero point at 7234 along the X-axis, with positive values on the left and negative values on the right. The treatment groups (LPS and LPS + Fraxin) were labeled accordingly in the enrichment plot. The positive ES and NES values supported significant enrichment of the PI3K-Akt pathway in the analyzed gene set (Figure 2D).

### 2.3. Biological Functions and Pathways Related to Apoptosis and Inflammation Were Identified by Enrichment Analysis

**Figure 3:**
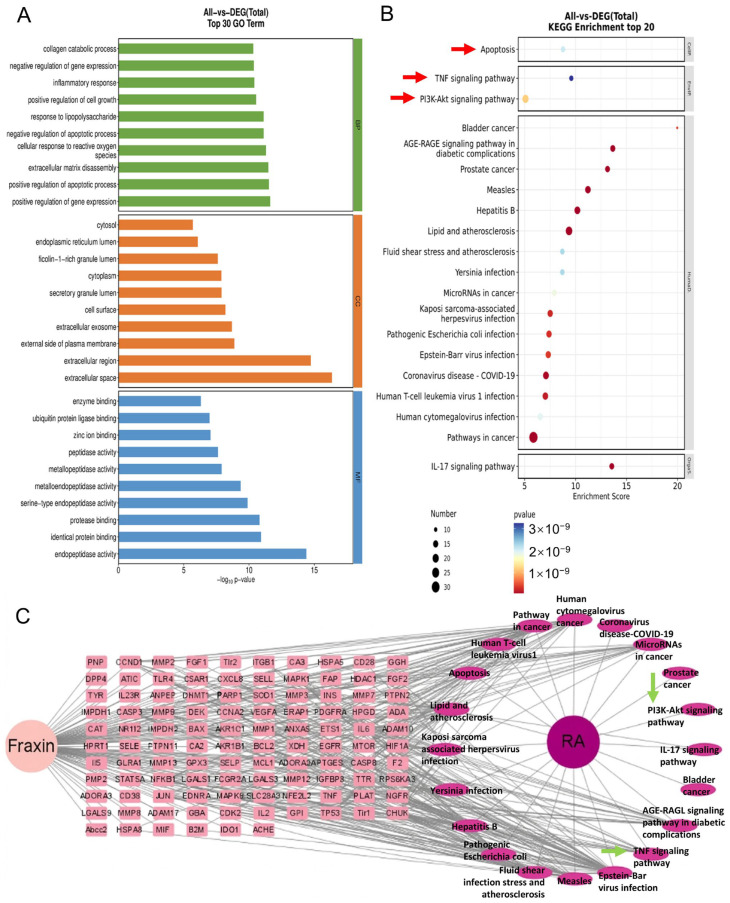
GO and KEGG enrichment analysis. (**A**) GO enrichment analysis. (**B**) KEGG enrichment analysis. (**C**) Fraxin-RA target-pathway enrichment network. The arrows indicate the pathways we selected.

GO analysis of the identified targets revealed significant involvement in several biological processes (BP), including collagen degradation, inflammatory responses, and apoptosis (Figure 3A). Molecular function (MF) terms indicated the enrichment in endopeptidase and metalloenzyme activities, while cellular component (CC) analysis showed associations with exocytosis and intracellular cytosolic localization. KEGG pathway analysis further identified the apoptosis pathway, TNF signaling pathway, and PI3K-Akt signaling pathway as major pathways potentially regulated by Fraxin (Figure 3B). Previous studies have demonstrated that PI3K/AKT and TNF-α pathways play important roles in regulating FLS apoptosis and joint inflammation [25,26,27,28]. Based on these findings, a compound–target–pathway–disease network was constructed, supporting the hypothesis that Fraxin may regulate apoptosis via the PI3K/Akt pathway and modulate FL-mediated inflammation through the TNF-α pathway (Figure 3C).

### 2.4. Molecular Docking and Molecular Dynamics Simulation Analysis of the HSPA8-Fraxin Interaction

**Figure 4:**
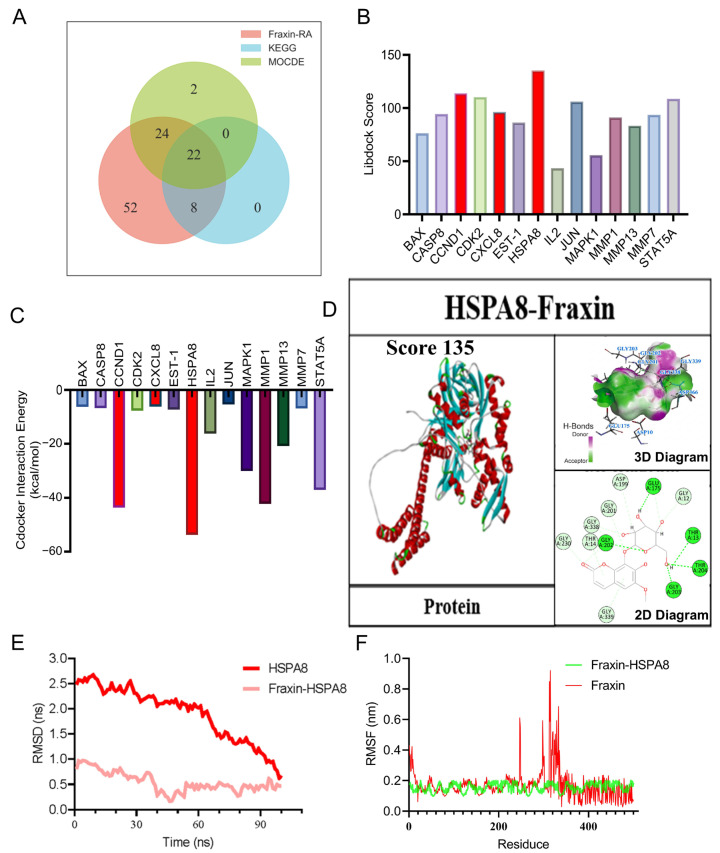
Molecular docking and molecular dynamics simulation analysis of HSPA8-Fraxin. (**A**) Venn diagram of intersection targets, targets obtained from the MOCED algorithm, and the important eight points obtained from NIM. (**B**) Statistics on interconnection results in Libdock mode. (**C**) Statistics on interconnection results in CDOCKER mode. (**D**) Schematic diagram of 2D and 3D docking results of HSPA8-Fraxin. (**E**) Values of root mean square deviation. (**F**) Values of root mean square.

To further clarify the molecular targets of Fraxin, 22 core candidates were identified through Venn diagram intersection analysis (Figure 4A). Molecular docking was performed using Discovery Studio 4.5 software under both Libdock and CDOCKER modules. The docking results were quantitatively evaluated and visualized (Figure 4B,C). Among the docking results, three complexes exhibited superior interaction properties based on bond type, interaction count, and bond distance. The Fraxin–HSPA8 complex showed a docking score of 135 and a binding energy of −53.7 kcal/mol. Detailed interaction analysis revealed specific active sites on HSPA8 for Fraxin binding (Figure 4D), indicating that HSPA8 may be a direct target of Fraxin. To further evaluate the stability of this interaction, a 100 ns molecular dynamics simulation was performed. Root mean square deviation (RMSD) analysis was used to assess the structural stability of Fraxin and HSPA8 throughout the simulation, and the results showed that the Fraxin–HSPA8 complex was more stable than unbound HSPA8 (Figure 4E). Root mean square fluctuation (RMSF) analysis demonstrated that most amino acid residues in the complex showed only limited fluctuation, with an average fluctuation of approximately 0.2 nm. However, residues in the 320–340 region exhibited relatively greater flexibility, which may reflect localized conformational dynamics during ligand binding (Figure 4F). Together, these results support a stable and specific interaction between Fraxin and HSPA8.

### 2.5. Fraxin Reduced Inflammatory Response in FLs and Macrophages

**Figure 5:**
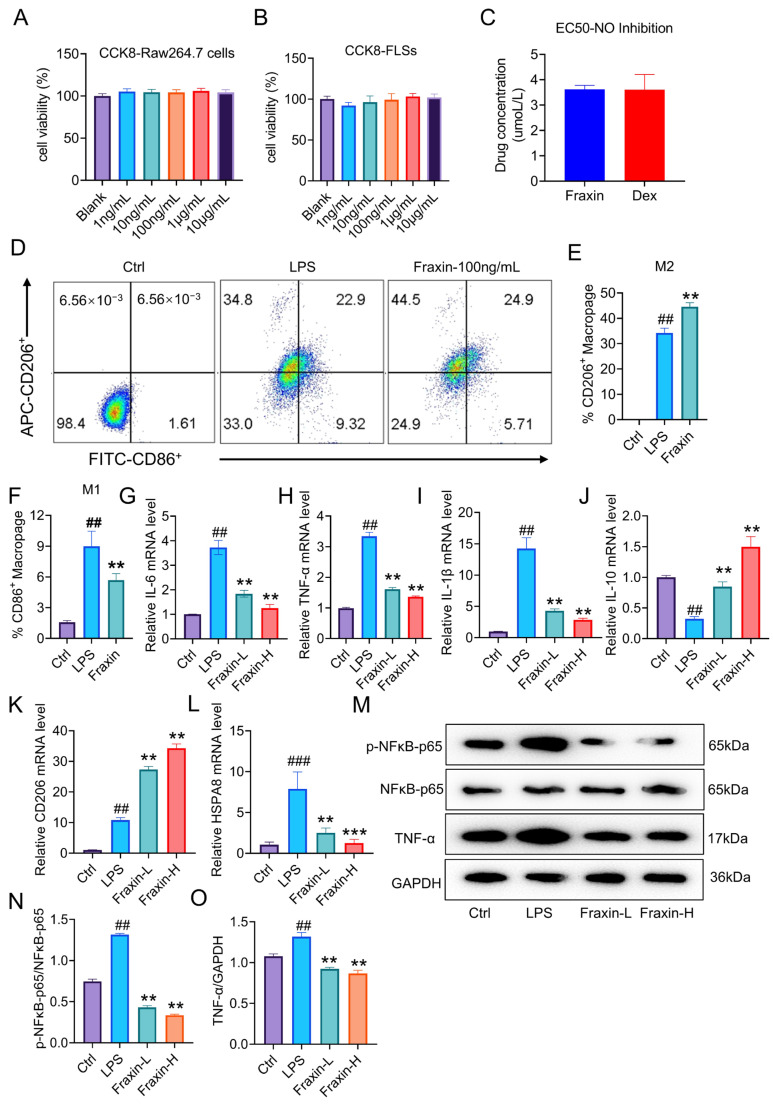
Fraxin inhibited inflammation in Raw264.7 cells and FLs. (**A**,**B**) Effect of Fraxin on the cellular viability of Raw264.7 and FLs. (**C**) EC-50 of Fraxin (100 ng/mL) in Raw264.7 cells. (**D**–**F**) Flow cytometry analysis showing the polarization effect of Fraxin (100 ng/mL) on Raw264.7 cells. (**G**–**L**) RT-PCR analysis of *Il6*, *Tnfα*, *Il1b*, *Il10*, *Cd206*, and *Hspa8* in FLs treated with Fraxin. (**M**–**O**) Protein levels of p-NFκB-p65 and TNF-α were examined by Western blot in FLs. Fraxin low-dose group (Fraxin-L, 100 ng/mL), Fraxin high-dose group (Fraxin-H, 1 μg/mL). ## *p* < 0.01, ### *p* < 0.001 versus Ctrl; ** *p* < 0.01, *** *p* < 0.01 versus LPS.

CCK-8 assays showed that Fraxin did not significantly affect the viability of RAW264.7 macrophages (Figure 5A) or FLs (Figure 5B). EC50 analysis further indicated that the effective concentration range of Fraxin was comparable to that of dexamethasone in FLs (Figure 5C). Flow cytometry revealed that Fraxin promoted macrophage polarization toward the M2 phenotype (Figure 5D–F). Real-time PCR analysis showed significant reductions in the mRNA expression of inflammatory cytokines *Il6*, *Tnfa*, and *Il1b* in Fraxin-treated cells (Figure 5G–I). Concurrently, levels of the anti-inflammatory cytokine *Il10* (Figure 5J) and *Cd206* (Figure 5K) were elevated. Notably, the mRNA (Figure 5L) and protein levels (Figure S1A,B) of *Hspa8* in FLs were also significantly downregulated by Fraxin. Also, Fraxin downregulated the protein and mRNA levels of HSPA8 in LPS-stimulated RAW264.7 cells (Figure S1C–E).

Activation of the TNF-α/NFκB signaling pathway is closely related to the development of RA [29]. Inhibitor of κB alpha (IκBα) plays a central role in regulating the NFκB signaling pathway [14,30]. In RA, TNF-α leads to persistent IκBα phosphorylation and degradation, resulting in sustained NFκB activation and chronic inflammation [31]. Western blot analysis showed that LPS stimulation markedly increased the protein levels of phosphorylated NF-κB p65 and TNF-α in FLSs, whereas Fraxin treatment significantly reduced both proteins (Figure 5M–O). In addition, LPS increased the phosphorylation of IκBα while decreasing total IκBα expression in FLSs (Figure S2A,B), and these changes were reversed by Fraxin treatment (Figure S2A,B). Consistently, in RAW264.7 cells, Fraxin suppressed NF-κB p65 phosphorylation, prevented IκBα phosphorylation and degradation, and reduced TNF-α protein expression after LPS challenge (Figure S3A,B). These findings suggest that Fraxin inhibits inflammatory responses in FLSs and macrophages, at least in part, by suppressing the IκBα/NF-κB/TNF-α signaling pathway.

### 2.6. Fraxin Improved RA Symptoms in Spontaneous IL1RA^−/−^ Mice

**Figure 6:**
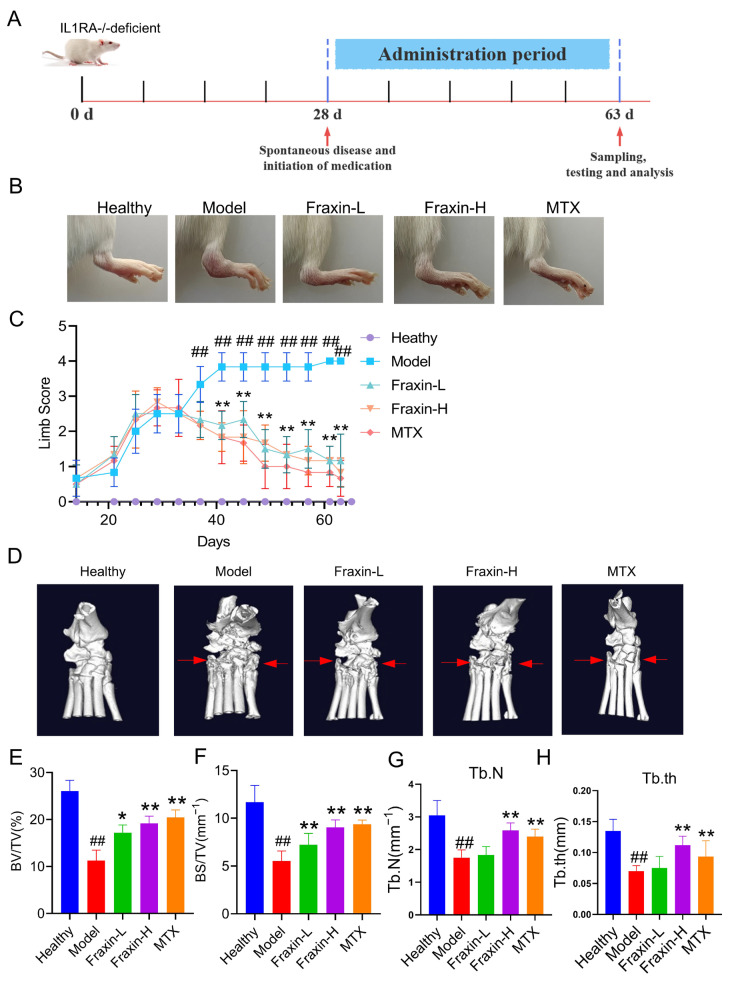
Fraxin improved RA symptoms in spontaneous IL1RA^−/−^ arthritis mice. (**A**) Animal experiment design. (**B**) Image of ankle swelling in spontaneous arthritis mice. (**C**) Ankle swelling score of spontaneous arthritis mice. (**D**) Micro-CT images of the ankle of spontaneous arthritis mice. The arrows indicate the joint lesion areas. (**E**–**H**) BV/TV, BS/TV, Tb.N and Tb.th of mice. Fraxin low-dose group (Fraxin-L, 10 mg/kg), Fraxin high-dose group (Fraxin-H, 30 mg/kg). IL1RA, Interleukin-1 Receptor Antagonist; BV, bone volume; TV, tissue volume; Tb.N, trabecular number; Tb.th, trabecular thickness; MTX, Methotrexate. ## *p* < 0.01 versus Ctrl; * *p* < 0.05 versus Model; ** *p* < 0.01 versus Model.

**Figure 7:**
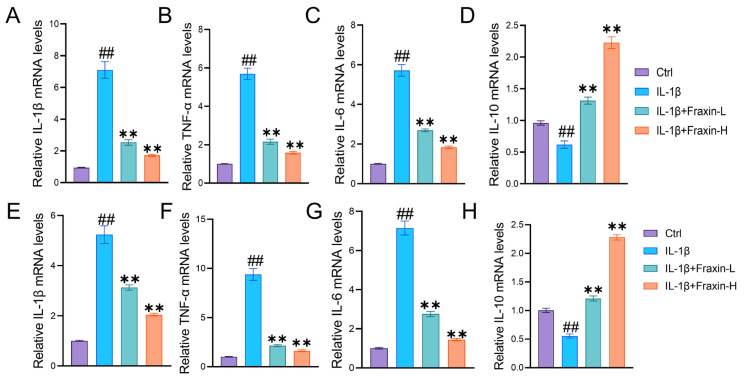
Fraxin inhibited inflammation in Raw264.7 cells and FLs. For treatment, Raw264.7 cells and FLs were pre-incubated with different doses of Fraxin (100 ng/mL or 1 μg/mL) for 30 min, followed by IL-1β stimulation (10 ng/mL) for 24 h. (**A**–**D**) RT-PCR analysis of *Il1b*, *Tnfa*, *Il6*, and *Il10* in IL-1β (10 ng/mL)-induced Raw264.7 cells treated with Fraxin. (**E**–**H**) RT-PCR analysis of *Il1b*, *Tnfa*, *Il6*, and *Il10* in IL-1β (10 ng/mL)-induced FLs treated with Fraxin. Fraxin low-dose group (Fraxin-L, 100 ng/mL), Fraxin high-dose group (Fraxin-H, 1 μg/mL). ## *p* < 0.01 versus Ctrl; ** *p* < 0.01 versus IL-1β.

IL1RA^−/−^ mice were used to evaluate the role of Fraxin in RA progression (Figure 6A). Mice in the model group exhibited typical RA symptoms, including joint swelling, erythema, and deformity. However, the foot swelling of mice in the Fraxin group was significantly relieved in a dose-dependent manner (Figure 6B). Limb scores indicated that Fraxin intervention alleviated disease progression (Figure 6C). Micro-CT analysis revealed severe ankle joint deformities in the model group, which were significantly improved by Fraxin and MTX treatment (Figure 6D). Further analysis using Analyze12.0 showed that BV/TV, BS/TV, Tb.N, and Tb.Th were markedly reduced in the model group. These changes were reversed by Fraxin treatment (Figure 6E–H). Notably, Fraxin and MTX produced comparable protective effects against experimental arthritis (Figure 6B–H). In addition, pretreatment with Fraxin significantly reduced the mRNA levels of Il1b, Tnfa, and Il6 in RAW264.7 cells after IL-1β stimulation (Figure 7A–C), while restoring Il10 expression (Figure 7D). Similar results were observed in FLs (Figure 7E–H). Together, these findings demonstrate that Fraxin effectively attenuates RA progression.

### 2.7. Fraxin Improved Synovial Hyperplasia

**Figure 8:**
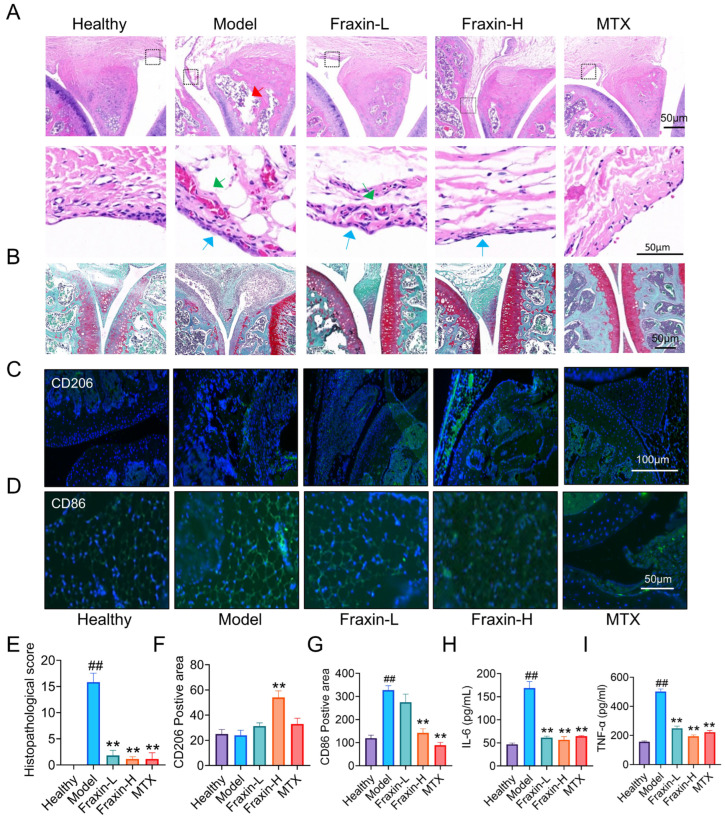
Fraxin improved the inflammatory microenvironment of the knee joint. (**A**) H&E staining of the knee joint. The area within the box represents the magnified region shown below. The red arrow points to meniscal injury, the blue arrow points to synovial thickening, and the green arrow points to synovial inflammatory pannus. (**B**) Knee joint staining with saffron solid green. (**C**,**D**) CD206 and CD86 immunofluorescence staining in the synovium. (**E**) The pathology score from H&E staining. (**F**,**G**) Quantitative analysis of CD206 and CD86 immunofluorescence staining. (**H**) Serum levels of IL-6 were analyzed by ELISA. (**I**) Serum levels of TNF-α were analyzed by ELISA. ## *p* < 0.01 versus Ctrl; ** *p* < 0.01 versus Model.

Histological examination of knee joints from the model group revealed typical RA features, including meniscal injury (red arrow), synovial thickening (blue arrow), and inflammatory pannus formation (green arrow). These pathological changes were ameliorated following Fraxin treatment, as indicated by H&E staining and corresponding histological scores (Figure 8A,E). Saffron-O and Fast Green staining showed pronounced cartilage degradation in the model group, which was repaired upon Fraxin administration (Figure 8B). Studies have shown that the imbalance of macrophage polarizations factors in local joint inflammation [32]. To assess this, M1 and M2 macrophages in the synovial tissue were detected using immunofluorescence staining. Fraxin-H treatment resulted in a marked increase in M2 macrophages (Figure 8C,F), while M1 macrophage levels were elevated in the model group. Both Fraxin-H and MTX significantly reduced M1 macrophage expression (Figure 8D,G). Additionally, ELISA analysis of serum TNF-α and IL-6 levels indicated strong inflammatory responses in the model group, both of which were reversed by Fraxin and MTX (Figure 8H,I). These findings suggest that Fraxin effectively improved the inflammatory microenvironment in RA joints.

### 2.8. Fraxin Regulated the Abnormal Proliferation of FLs

**Figure 9:**
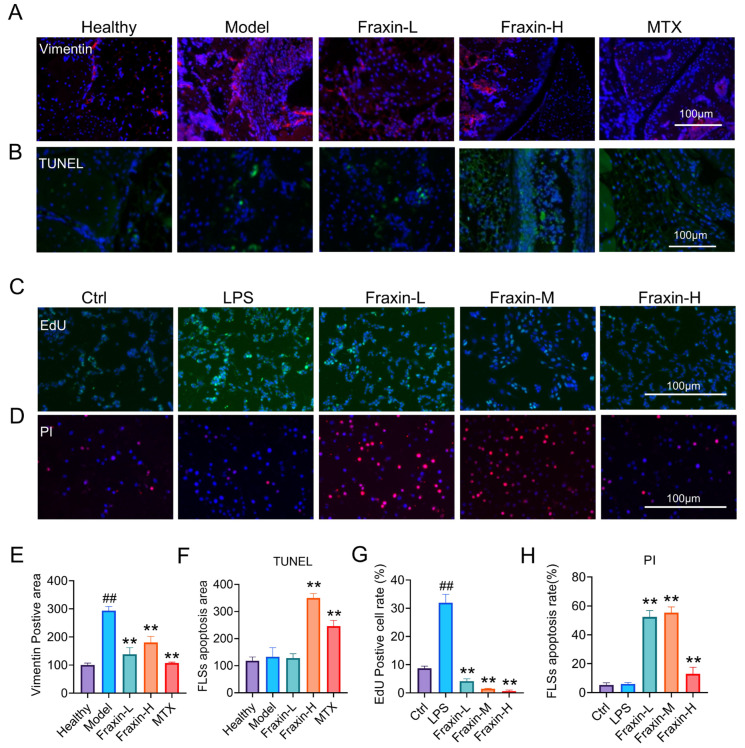
Fraxin promoted apoptosis of FLs. (**A**) Immunofluorescence image of FLS marker vimentin in the synovium. (**B**) TUNEL staining in the synovium. (**C**) EdU assay was used to detect the proliferation of FLs in vitro. (**D**) PI staining showing the apoptosis of FLs. (**E**) Quantitative analysis of vimentin-positive areas. (**F**) Quantitative analysis of FLS apoptosis in the synovium. (**G**) Quantitative analysis of EdU-positive cells. (**H**) Quantitative analysis of FLS apoptosis in cells. ## *p* < 0.01 versus Ctrl; ** *p* < 0.01 versus Model or LPS.

Immunofluorescence staining for vimentin, a marker of FLs in the knee joint synovium, revealed significant FLS proliferation in the model group, which was inhibited by Fraxin treatment (Figure 9A,E). TUNEL staining of synovial tissue showed that Fraxin reduced abnormal hyperplasia by promoting apoptosis of FLs (Figure 9B,F). Similarly, EdU staining (Figure 9C,G) and PI (Figure 9D,H) staining further supported these findings, demonstrating that Fraxin inhibited cell proliferation and enhanced apoptosis in FLs. These findings suggest that Fraxin effectively inhibits the abnormal proliferation of FLs.

### 2.9. Fraxin Induced FLs Mitochondrial Membrane Potential Dysregulation Through HSPA8

**Figure 10:**
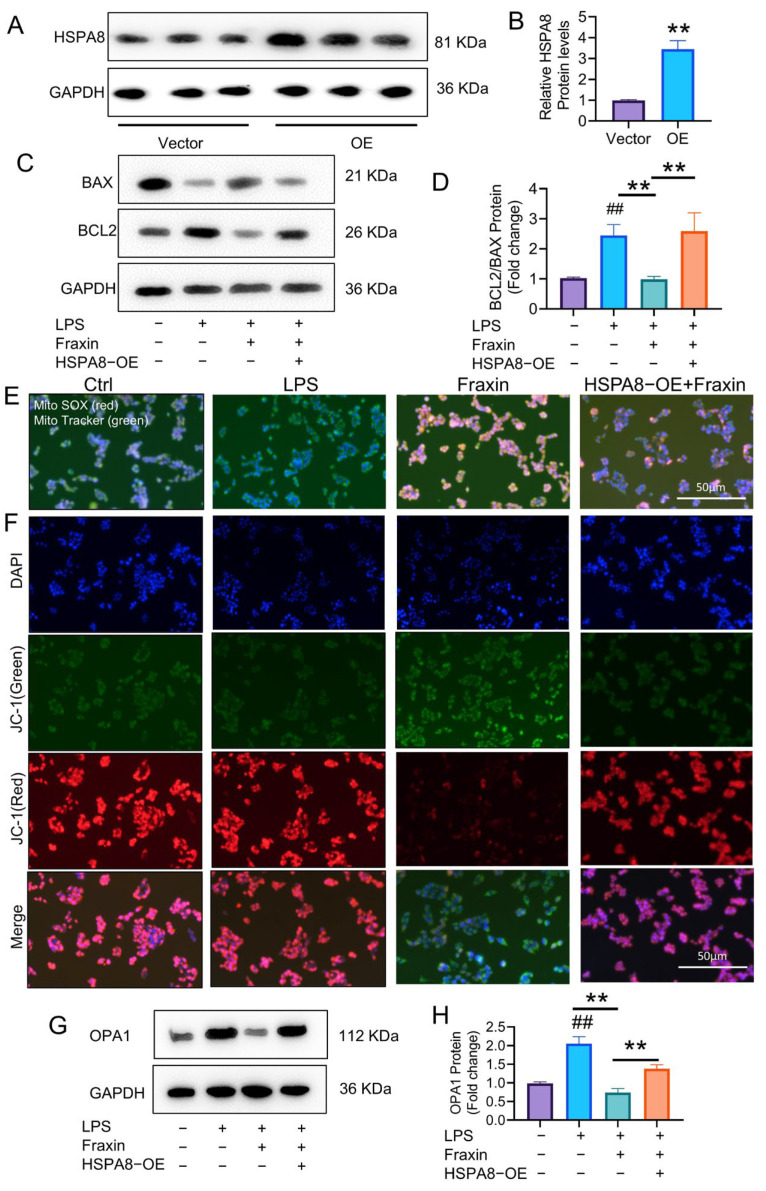
Fraxin induced mitochondrial membrane potential dysregulation through inhibiting HSPA8 in FLs. (**A**,**B**) The protein expression of HSPA8. (**C**,**D**) Detection of BAX and BCL2 protein expression levels. (**E**) Mito SOX (red) and Mito Tracker (green) co-staining of FLs. (**F**) JC-1 staining. (**G**,**H**) Detection of OPA1 protein expression. ## *p* < 0.01 versus Vector or Ctrl; ** *p* < 0.01 versus LPS.

Mitochondrial dysfunction is a key factor in the generation of excessive reactive oxygen species (ROS), which disrupts cellular homeostasis and trigger apoptosis, contributing to various human diseases, including RA. To investigate whether Fraxin promotes FL apoptosis by inducing mitochondrial dysfunction via HSPA8, we constructed plasmids to overexpress HSPA8 in FLs (Figure 10A,B). At the protein level, Fraxin significantly reduced the ratio of BCL2/BAX expression, but this effect was reversed by HSPA8 overexpression (Figure 10C,D). MitoSox (red) and Mito Tracker (green) staining showed that Fraxin potentiated the production of mitochondrial ROS in FLs. However, this was significantly reversed by HSPA8 overexpression (Figure 10E). In healthy mitochondria, the 5,5′,6,6′-Tetrachloro-1,1′,3,3′-tetraethylbenzimidazolylcarbocyanine iodide (JC-1) dye accumulates in its aggregate (red) form, whereas in dysfunctional mitochondria, JC-1 shifts to its monomeric (green) form due to the loss of mitochondrial membrane potential (MMP). In the Fraxin-treated FLs, mitochondria showed more green signals, indicating lower MMP, but HSPA8 overexpression restored the red signal, indicating recovery of mitochondrial function (Figure 10F). Optic Atrophy 1 (OPA1), which is critical for maintaining mitochondrial cristae structure, was also downregulated by Fraxin in FLs. HSPA8 overexpression reversed this downregulation (Figure 10G,H). These results suggest that Fraxin promotes mitochondrial defect-mediated apoptosis in FLs via the HSPA8 pathway.

### 2.10. Fraxin Reduced the Proliferation of Migration of FLs by Inhibiting the HSPA8/PI3K Pathway

Overexpression of HSPA8 reversed the inhibitory effects of Fraxin on PI3K/AKT activation and TNF-α expression in LPS-stimulated FLSs (Figure S4A,B). In addition, the anti-inflammatory effects of Fraxin were attenuated by HSPA8 overexpression (Figure S4C). Consistently, the pro-apoptotic effect of Fraxin on FLSs, as detected by PI staining, was also abolished by HSPA8 overexpression (Figure S4D).

**Figure 11:**
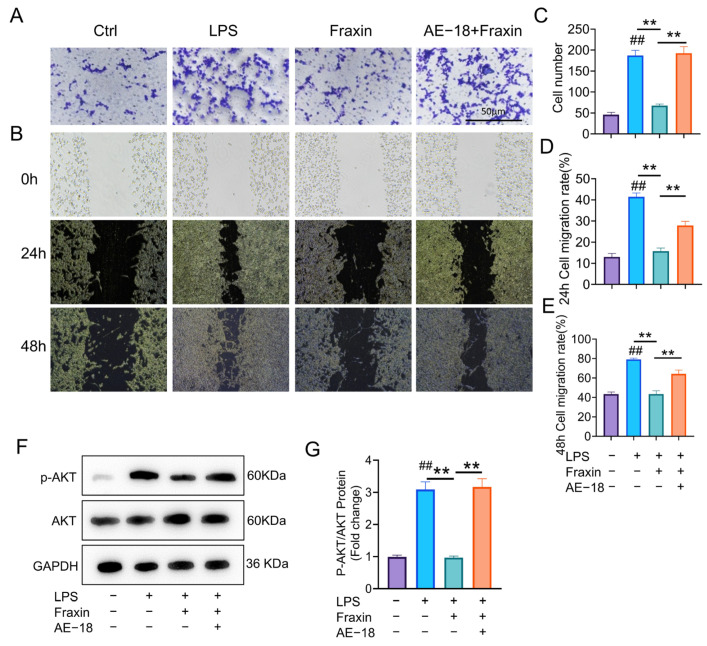
Fraxin reduced the migration of FLs by inhibiting the PI3K/AKT pathway. (**A**) Transwell experiment of FLs. (**B**) Scratch test of FLs. (**C**) Quantitative analysis of the migration number of FLs. (**D**,**E**) Quantitative analysis of the migration rate of FLs at 24h and 48h. (**F**,**G**) The protein expression of p-AKT. ## *p* < 0.01 versus Ctrl; ** *p* < 0.01 versus LPS.

Transwell and scratch assay showed that Fraxin significantly inhibited the invasion and migration of LPS-induced FLs. However, this phenomenon was significantly reversed when the PI3K agonist AE-18 was present (Figure 11A–E). The protein expression of p-AKT was downregulated in FLs after treatment with Fraxin, an effect that was reversed by the PI3K agonist AE-18 (Figure 11F,G). Likewise, AE-18 pretreatment abolished the effects of Fraxin on TNF-α protein expression (Figure S5A), inflammation (Figure S5B), and apoptosis (Figure S5C) in LPS-exposed FLs. These findings collectively indicate that Fraxin exerts anti-inflammatory and pro-apoptotic effects on FLs by targeting the HSPA8/PI3K/AKT axis, thereby reducing TNF-α expression and suppressing synovial hyperplasia in RA.

## 3. Discussion

RA is a multifactorial chronic autoimmune disease influenced by environmental, dietary, and genetic factors. In its early stages, RA typically presents as joint stiffness and pain caused by inflammation of the synovial tissue. Without timely intervention, the disease progresses to irreversible joint damage and loss of function. Existing research has shown that Fraxin has strong anti-inflammatory activity [33,34]. It has been established that Fraxin inhibits chondrocyte apoptosis, thereby preventing knee osteoarthritis and emerging as a promising candidate for anti-osteoarthritis therapy [21] A total of 9 potential active constituents, including kaempferol, quercetin, naringenin, paeoniflorin, catechin, fraxin, gentianin, hesperetin, and ellagic acid 3,3’,4-trimethyl ether, might contribute to the effects of Semiliquidambar cathayensis Chang Roots on reducing arthritis score and alleviating ankle joint histopathology in RA rats [35]. Despite these observations, the roles and mechanisms of Fraxin in RA remain unclear. Therefore, in this study, we evaluated the therapeutic effect of Fraxin on RA in vivo and in vitro. In vivo, Fraxin significantly inhibited foot swelling, bone deformities, and improved bone volume fraction in IL1RA^−/−^ mice. In vitro, Fraxin significantly inhibited the expression of HSPA8 in LPS-exposed FLs. Mechanistically, Fraxin inactivated the PI3K/AKT and IκBα/NFκB/TNF-α signaling pathways in macrophages and FLs. In addition, Fraxin inhibited synovial cell hyperplasia by regulating mitochondrial membrane potential collapse in FLs. Overall, these findings suggest that Fraxin may mitigate arthritis progression by modulating mitochondrial function and stress-related pathways in FLs, ultimately reducing joint inflammation and bone destruction. While Fraxin showed significant therapeutic effects in preclinical models, there is currently no clinical data to support its use in human RA patients. Future research should aim to transition these findings into clinical trials to assess the therapeutic potential of Fraxin in a clinical setting. Such trials should investigate the optimal dosage, formulation, and delivery methods, as well as the interactions of Fraxin with current standard-of-care treatments for RA, such as DMARDs and biologics.

Chinese herbal formulas have long been used in TCM to treat a variety of chronic inflammatory diseases, including RA. Current RA treatment mainly relies on DMARDs and biologic agents. Although these therapies are effective, their adverse effects and long-term risks have increased interest in complementary strategies such as Chinese herbal medicine. For example, mangiferin and cinnamic acid, the major active ingredients of Baihu-Guizhi decoction, have been shown to alleviate RA by inhibiting the TLR4/NF-κB/NLRP3 pathway [36]. Duhuo Jisheng pill alleviates CIA through inhibiting the PI3K/AKT/NF-κB signaling pathway [37]. Fraxin has strong anti-inflammatory activity in several disease settings [33], including renal dysfunction [38], diabetes nephropathy [39], and osteoarthritis [40]. Our findings further extend these observations and support the potential therapeutic value of Fraxin in RA.

Mitochondrial dysfunction is increasingly recognized as a contributor to RA pathogenesis through oxidative stress-mediated inflammatory signaling [41,42,43]. Mitochondria also regulate apoptosis, a process critical to the maintenance of synovial tissue homeostasis [44,45]. In this study, Fraxin was shown to induce mitochondrial membrane potential dysregulation in FLs through a mechanism involving HSPA8. The construction and transfection of an HSPA8 overexpression plasmid demonstrated that upregulation of HSPA8 could reverse the apoptosis induced by Fraxin, suggesting that HSPA8 is a key molecular target of Fraxin in RA. Furthermore, Fraxin inhibited FLS migration by suppressing the PI3K/HSPA8 signaling pathway. Although our present study identified HSPA8 as a target of Fraxin based on docking score, binding energy, and interaction-site analysis, the in silico results also suggest potential interactions of Fraxin with other molecules, such as IL-8 and CCND1, which may be biologically relevant in the context of RA. Specifically, IL-8 is a potent leukocyte chemoattractant that contributes to inflammatory cell recruitment and amplification of synovial inflammation in RA [46], whereas CCND1 is closely associated with the regulation of synovial fibroblast proliferation and apoptosis [47], both of which are critical processes in pannus formation and joint destruction. Given the multifactorial nature of RA, these observations support the possibility that Fraxin exerts its therapeutic effects through multiple targets and signaling pathways rather than a single molecular axis. Further experimental studies are needed to verify the biological relevance of these predicted interactions, particularly those involving IL-8 and CCND1.

The activation of the PI3K/AKT pathway is closely related to the development of RA [48]. Shikonin inhibits angiogenesis and alleviates arthritis by downregulating the PI3K/AKT pathway [49]. Magnoflorine alleviates the inflammatory response of RA by regulating the PI3K/Akt/NF-κB signaling pathway [50]. Our KEGG analysis identified several signaling pathways associated with RA. Among these, we focused on the apoptosis signaling pathway, TNF-α pathway, and PI3K/AKT pathway. Although the enrichment scores of these three pathways were not the highest, they were prioritized due to their well-documented roles in RA pathogenesis. For instance, the PI3K/AKT pathway is crucial for the proliferation and survival of FLs [51], while the TNF-α pathway is a central mediator of synovial inflammation [52]. Furthermore, apoptosis dysregulation is a hallmark of synovial hyperplasia in RA [53]. Based on these findings and previous literature, we constructed a drug–target–pathway–disease network and proposed that Fraxin regulates FLS function by modulating the PI3K/AKT and TNF-α signaling pathways.

To further explore the relationship between HSPA8 and PI3K signaling, we used the PI3K agonist AE-18 in cellular experiments. Transwell and wound-healing assays showed that AE-18 reversed the inhibitory effects of Fraxin on FLS migration and invasion. This pattern was similar to that observed after HSPA8 overexpression. Although the present study focused mainly on apoptosis, TNF-α, and PI3K/AKT signaling because of their clear relevance to RA pathogenesis, we acknowledge that other pathways showed even higher enrichment scores in the KEGG analysis. These included pathways related to AGE-RAGE signaling, lipid metabolism, and IL-17 signaling, all of which are also implicated in joint inflammation and tissue remodeling in RA. Future studies should therefore investigate whether Fraxin modulates these highly enriched pathways, either independently or in concert with PI3K/AKT and TNF-α signaling. Such work may include targeted transcriptomic or proteomic analyses, pathway-specific intervention experiments, and validation in additional in vivo RA models. This broader approach may help to clarify the full signaling network through which Fraxin exerts its therapeutic effects.

Previous studies have highlighted the importance of synovial tissue as a site of inflammation and pathological remodeling in RA [54,55]. Within synovial tissue, FLSs are the predominant stromal cell type and directly contribute to abnormal proliferation, migration, and invasion. Consistent with this concept, our study showed that Fraxin effectively inhibited FLS migration and invasion through regulation of HSPA8. Notably, overexpression of HSPA8 reversed the effects of Fraxin on PI3K/AKT activation, TNF-α expression, inflammation, and apoptosis in FLSs. These findings indicate that HSPA8 plays a critical role in mediating the action of Fraxin and may function as an upstream regulator of PI3K/AKT signaling in RA.

Our data further support the view that the HSPA8/PI3K/AKT axis is a key signaling pathway underlying the effects of Fraxin in FLSs. Overexpression of HSPA8 reversed the inhibitory effects of Fraxin on PI3K/AKT activation, TNF-α expression, and synovial cell hyperplasia, while PI3K activation by AE-18 abolished the anti-inflammatory and pro-apoptotic effects of Fraxin. These results suggest that the HSPA8/PI3K/AKT axis is a major signaling pathway mediating the action of Fraxin. Although apoptotic signaling and TNF-α expression were clearly altered by Fraxin, these changes are likely downstream consequences of HSPA8/PI3K/AKT modulation rather than fully independent mechanisms. Nevertheless, crosstalk between HSPA8/PI3K/AKT and other signaling pathways cannot be excluded. Further genetic and pharmacological studies are required to determine whether Fraxin regulates these pathways sequentially or in parallel during RA progression.

## 4. Conclusions

In conclusion, Fraxin was identified from WGL using UPLC-Q-TOF-MS and serum untargeted metabolomics. Network pharmacology screening revealed that Fraxin alleviates RA progression by targeting HSPA8. GO and KEGG enrichment analyses confirmed that Fraxin exerts its regulatory effects through the PI3K signaling pathway. The therapeutic role of Fraxin was validated both in a spontaneous arthritis mouse model and in LPS-induced FLs. Functional studies demonstrated that Fraxin regulates mitochondrial collapse-mediated apoptosis in FLs via the PI3K/HSPA8 pathway, thereby inhibiting excessive FLS proliferation and migration and slowing the progression of RA. Despite these promising findings regarding the anti-inflammatory and anti-RA effects of Fraxin, several limitations should be noted. Although the use of RAW 264.7 cells, FLs, and the IL1RA^−/−^ spontaneous arthritis model provided mechanistic insights, these systems do not fully capture the complexity of human RA. Further studies should investigate Fraxin’s effects in more representative models, including patient-derived cells or humanized RA mouse models, to improve translational relevance. In addition, the current study focused mainly on the short-term effects of Fraxin, particularly on apoptosis and mitochondrial function in FLs. However, long-term safety and efficacy data, including assessments of toxicity, immunogenicity, and potential side effects, remain unavailable. Clinical trials and extended preclinical evaluations are necessary to determine whether Fraxin can be safely applied in human RA, especially given the chronic nature of the disease and the need for sustained therapeutic intervention. In addition, no human data were included in the present study, and thus the translational relevance of our findings requires more comprehensive validation in clinical samples. The IL1RA^−/−^ spontaneous arthritis mice used here might represent an acute form of the disease, which may not fully recapitulate the chronic and heterogeneous nature of human RA. Although our results support the therapeutic potential of Fraxin, the possibility of off-target effects cannot be excluded, and further mechanistic studies are highly warranted. Finally, only male IL1RA^−/−^ mice were included in this study. As RA is more prevalent in women [56] and sex-related differences may influence disease pathogenesis and drug responses, caution is needed when extrapolating these findings. Future studies should incorporate female mice to validate the generalizability of the results. The extracellular vesicles (EVs) play a pivotal role in intercellular communication and immune regulation via transferring bioactive molecules, such as non-coding RNAs (ncRNAs) [57]. Recent studies have highlighted the important role of EVs in the pathogenesis of RA [58]. In particular, EVs derived from patients with RA can promote dendritic cell activation and contain modified autoantigens, including citrullinated and carbamylated proteins, thereby enhancing autoimmune activation and synovial inflammation [59]. These findings suggest that EVs-mediated intercellular communication is an important component of RA progression. Although the present study did not directly investigate the influence of Fraxin on EVs-associated pathways, its anti-inflammatory and anti-rheumatic effects may also involve modulation of EV release, cargo composition, or EVs-mediated immune activation. Further studies are needed to determine whether regulation of EV signaling contributes to the therapeutic effects of Fraxin in RA.

## 5. Materials and Methods

### 5.1. Reagents and Chemicals

Fraxin (B10713) was purchased from Shanghai Biotechnology Co., LTD (Shanghai, China). SYBR Green Mix (11202ES03) was obtained from Yeasen Biotechnology Co., LTD (Shanghai, China). CD206 (A02285-2) and CD86 (BM4121) were purchased from Boster Biological Technology Co., LTD (Wuhan, China). CCK-8 (KTA1020) and EdU (KTA2030) were purchased from Abbkine Scientific Co., LTD (Wuhan, China). FreeZol Reagent (R711-01) was obtained from Nanjing Vazyme Biotechnology Co., LTD (Nanjing, China). F4/80(E-AB-F0995P) was obtained from Wuhan Elabscience Biotechnology Co., LTD (Wuhan, China). TNF-α, IL-6 ELISA Kit (MM-47831M2, MM-1011M2) were purchased from Jiangsu Meimian industrial Co., LTD (Yancheng, China). Mito-Tracker Green (C1048), Hoechst (C1011), JC-1(C2006) dye and BSA (ST025) were purchased from Beyotim Biotechnology Co., LTD (Shanghai, China). Modified Saffron-O and Fast Green Stain dye (G1371) was obtained from Beijing Solarbio Technology Co., LTD (Beijing, China). Lipopolysaccharide (LPS) from *Escherichia coli* O111:B4 (Sigma-Aldrich, St. Louis, MO, USA) was used for in vitro experiments. Other conventional reagents are purchased from Wuxi Weiwo Biosharp Biotechnology Co., LTD (Wuxi, China).

### 5.2. Screening of Potentially Active Substances

Using UPLC-Q-TOF-MS, the chemical constituents of the WGL compound and its five individual herbal components were analyzed. Total ion chromatograms in both positive and negative ion modes were generated. Compounds with significantly higher relative ion abundance in the compound mixture compared to individual herbs were selected. These were further filtered by blood component analysis, and anti-inflammatory candidates were identified based on SuperClass classification and supporting literature.

### 5.3. Drug Target Prediction

Drug targets for Fraxin were predicted using several online databases, including SwissTargetPrediction (http://swisstargetprediction.ch/, 2019 version, URL (accessed on 10 April 2024)), HERB (http://herb.ac.cn/, 2.0 version, URL (accessed on 10 April 2024)), PharmMapper (http://lilab-ecust.cn/pharmmapper/, 2024 version, URL (accessed on 10 April 2024)), SEA (http://sea.bkslab.org/, 1.0 version, URL (accessed on 10 April 2024)), and SuperPred (https://prediction.charite.de, 3.0 version, URL (accessed on 10 April 2024)). Targets retrieved from these databases using “Fraxin” as the keyword were merged for further analysis.

### 5.4. Disease Target Query

Disease-related targets were identified using OMIM (https://www.omim.org/, M47.1 version, URL (accessed on 14 April 2024)), DrugBank (https://go.drugbank.com/, 13.0 version, URL (accessed on 14 April 2024)), GeneCards (https://www.genecards.org/, V5.21, URL (accessed on 14 April 2024)), and PharmGKB (https://www.pharmgkb.org/, version and URL (accessed on 14 April 2024)) databases. “Rheumatoid arthritis” was used as the keyword for each search. All retrieved targets were combined, and duplicates were removed.

### 5.5. Target Library of Fraxin in RA and Construction of Venny Diagram

The predicted target library of Fraxin and the RA-related target library were intersected to identify potential targets of Fraxin in RA. A Venn diagram was generated using the online Venny tool (https://bioinfogp.cnb.csic.es/tools/venny/, 2.1.0 version, 14 April 2024) to visualize the overlapping targets.

### 5.6. Gene Enrichment (GO) Analysis

GO analysis of the overlapping targets was performed using the Meta database (version and URL (accessed on 24 April 2024)), with results categorized into BP, MF, and CC domains. KEGG pathway enrichment analysis was then conducted to identify relevant signaling pathways and their associated targets. Using the MOCDE target correlation algorithm, the most strongly correlated targets were identified. A combined analysis of KEGG enrichment scores and algorithmic correlation scores was used to extract the top 30 targets. These targets, along with the overlapping target set, were compared to determine the most relevant potential therapeutic targets of Fraxin in RA.

### 5.7. Molecular Docking

The 3D structure of Fraxin (PubChem CID, 5273568) in SDF format was downloaded from Pubchem database (https://pubchem.ncbi.nlm.nih.gov/compound/5273568, version and URL (accessed on 24 April 2024)), and the 3D structure of target proteins including BAX (PDB No. 8EJA), CASP8 (PDB No. 2LR8), CCND1 (PDB No. 9CSK), CDK2 (PDB No. 1FQV), CXCL8 (PDB No. 6QJB), EST-1 (PDB No. 4JVM), HSPA8 (PDB No. 1YUW), IL2 (PDB No. 3QB1), JUN (PDB No. 6MXQ), MAPK1 (PDB No. 6SLG), MMP1 (PDB No. 8WOL), MMP13 (PDB No. 2OW9), MMP7 (PDB No. 2Y6C), and STAT5A (PDB No. 1Y1U) in PDB protein database was queried from Uniprot database. They were respectively input into the Discovery Studio 2019 software (v19.1.0.18287, URL (accessed on 24 April 2024)) developed by BONIA, and Fraxin was defined as the ligand and the target protein as the receptor. The protein was further analyzed and the appropriate site radius was selected. Two modes of Libdock and CDOCKER were selected and ligand drugs were used to dock with receptor proteins. Libdock score and CDOCKER Interaction Energy are obtained. Based on the comprehensive evaluation of Libdock score and CDOCKER Interaction Energy, the TOP3 target proteins with the best binding stability were obtained.

### 5.8. Cell Culture and Cell Viability Assay

Mouse cell line FLs were obtained from Procell system (CP-M323, Wuhan, China) and cultured in DMEM supplemented with 10% FBS and 1% PS in a 5% CO_2_ incubator (Heracell VIOS 160i, Thermo Scientific, Waltham, MA, USA) at 37 °C [60]. RAW 264.7 macrophages (CL-0190, Procell system, Wuhan, China) were cultured in DMEM medium (Gibco, Grand Island, NY, USA) supplemented with 10% FBS and 1% penicillin/streptomycin at 37 °C in a 5% CO_2_ incubator. Cells in the logarithmic growth phase were collected for subsequent experiments, including passaging and stimulation. Cell numbers were determined using a hemocytometer. For treatment, cells were pre-incubated with different doses of Fraxin (0–10 μg/mL) for 30 min, followed by LPS stimulation (1 μg/mL) for 24 h. To investigate the role of the PI3K signaling pathway, FLs were pre-treated with AE-18 (10 μM) for 30 min prior to LPS exposure (1 μg/mL, 24 h) [61]. For the measurement of cellular viability, FLs and RAW264.7 cells in the logarithmic growth phase were harvested, digested, and resuspended in complete medium to a density of 2 × 10^4^ cells/well. Cells were seeded into 96-well plates and treated with different concentrations of Fraxin, followed by incubation at 37 °C with 5% CO_2_ for 24 h. After incubation, the culture medium was removed, and CCK-8 solution (Abbkine Scientific Co., Ltd, Wuhan, China) was mixed with fresh medium at a 1:9 ratio. Then, 100 µL of the mixture was added to each well. After 1 h of incubation, the absorbance at 450 nm was measured using a microplate reader.

### 5.9. RT-qPCR

LPS at a concentration of 1 μg/mL was used to stimulate FLs and Raw 264.7 cell for 24 h, and the drug Fraxin was given at a concentration of 100ng/mL for intervention. After 24 h, the samples were collected by TRIzol (Beyotim Biotechnology Co., LTD, Shanghai, China), and the total RNA was extracted from the cells in the different groups. After measuring the concentration, the total RNA was reversed to 4 μg cDNA by reverse transcriptase. SYBR green MIX was used to add cDNA and corresponding primer sequences into the system, and the relative expression of each mRNA was calculated by quantitative CT according to fluorescence. The primer sequences for target genes were provided as follows: *Il6*, 5’-GCCTTCTTGGGACTGATGCT-3’ (Forward), 5’TGTGACTCCAGCTTATCTCTTGG-3’ (Reverse); *Il1b*, 5’-TGCCACCTTTTGACAGTGATG-3’ (Forward), 5’-GGAGCCTGTAGTGCAGTTGT-3’ (Reverse); *Tnfa*, 5’-ACCCTCACACTCACAAACCA-3’ (Forward), 5’-ACCCTGAGCCATAATCCCCT-3’ (Reverse); *Il10*, 5’-GCTCCAAGACCAAGGTGTCT-3’ (Forward), 5’-AGGACACCATAGCAAAGGGC-3’ (Reverse); *Cd206*, 5’-AGGGAAGAGAAGAAGATCCA-3’ (Forward), 5’-TTGACTTCATCTTCTCCCAG-3’ (Reverse); *Hspa8*, 5’-CAAGAGAGCUGUCCGCCGU-3’ (Forward), 5’-AGUCACAGAUCCAUGAUAU-3’ (Reverse); *β-actin*, 5’-ATGCCCTGAGGCTCTTTTCC-3’ (Forward), 5’-CAGCTCAGTAACAGTCCGCC-3’ (Reverse).

### 5.10. Transcriptome Sequencing

Transcriptome sequencing was performed by OE Biotechnology Co., LTD (Shanghai, China). Briefly, RNA was extracted from the samples using TRIzol reagent (Invitrogen, Carlsbad, CA, USA). RNA purity and concentration were assessed using a NanoDrop spectrophotometer (Thermo Scientific, USA) and an Agilent 2100 Bioanalyzer (Agilent Technologies, Santa Clara, CA, USA) to assess RNA integrity. RNA libraries were prepared using the VAHTS Universal V6 RNA-Seq Library Preparation kit. Sequencing was performed on an Illumina NovaSeq 6000 platform using 150 bp paired-end reads. Raw reads in fastq format were processed with fastp1 to remove low-quality reads and obtain clean reads. After filtration, clean readings from each sample were retained for further analysis. Clean reads were aligned to the reference genome using HISAT22. Gene expression was quantified by calculating FPKM values for each gene, and gene read counts were obtained using HTSeq-count4. Hierarchical clustering of DEGs was performed in R to show gene expression patterns between different groups and samples. The gradar package in R (v 3.2.0) software was used to generate radar maps of the top 30 up-regulated and down-regulated DEGs.

### 5.11. Animal Model Construction

Male IL-1β receptor antagonist knockout (IL1RA^−/−^) mice under BALB/c background were purchased from Jiangsu GemPharmatech Co., LTD (Nanjing, China). The IL1RA^−/−^ mice spontaneously develop severe polyarthritis starting at around 6 weeks of age, serving as a classic model for RA research. Agarose nucleic acid gel electrophoresis was used to identify the mouse genotype. Brief, a total of 70 mg tail tissue from a mouse aged 2 weeks old. An appropriate amount of digestion buffer and Proteinase K solution were added and incubated in a 55 °C water bath overnight. After centrifugation at 12,000 rpm for 1 min, 450 µL of the supernatant was collected, and 900 µL of absolute ethanol was then added. The wells were mixed and centrifuged again for 15 min, the supernatant was discarded, and the pellet was washed with 70% ethanol. The ethanol was discarded, and the pellet was dissolved in deionized water. The primers were added and the DNA was amplified using the Mouse Genotyping Kit (Beyotim Biotechnology Co., LTD, Shanghai, China. Then, the samples were loaded onto an agarose gel for electrophoresis. The mice were raised in the SPF mice feeding environment of Wuxi Medical College of Jiangnan University. Two weeks after breeding, the mice were sampled for genetic identification, and the homozygous mice were selected as the model group without intervention. Male BALB/c-background wild-type and IL1RA^−/−^ mice, aged 4 weeks at the start of treatment, were used in this study.

### 5.12. Animal Administration and Arthritis Score

The animals were divided into five groups, including a normal control group, a model group, Fraxin low-dose group (Fraxin-L, 10 mg/kg), Fraxin high-dose group (Fraxin-H, 30 mg/kg), and MTX positive drug group (0.8 mg/kg), with 8 mice in each group. During the treatment period, the mice in the Fraxin-H and Fraxin-L groups were administered Fraxin daily by oral gavage at the indicated doses. MTX was administered to mice by oral gavage every day at the indicated doses. All animal experiments were approved by the Experimental Animal Ethics Committee of Jiangnan University (Approval Code: JN.No20240630m0301015, Approval Date: 30 June 2024). They were housed in the SPF level environment of the experimental animal center of Wuxi Medical College of Jiangnan University, with the temperature controlled at 22–24 °C, the humidity controlled at 40–70%, normal diet and drinking water, and the ethics batch number of experimental animals. The degree of foot swelling in mice was clinically scored every 4 days after the start of administration. The foot state of mice was photographed and recorded after 5 weeks of administration. The clinical scoring criteria were: no erythema and swelling (0 point); erythema and mild swelling of ankle joint (1 point); Erythema and mild swelling from ankle to ankle, 2 points; Erythema and moderate swelling from ankle to metatarsal joint, 3 points; Erythema and severe swelling of ankles, toes or limbs, 4 points [62].

### 5.13. Micro-CT

In accordance with animal welfare guidelines, the ankle and plantar regions of each mouse were scanned using the ultra-high-resolution mode of Micro-CT. Following scanning, three-dimensional reconstruction was performed at 18 μm resolution. Bone structural parameters were analyzed using Analyze 12.0 software.

### 5.14. Hematoxylin-Eosin Staining

Mouse knee joints were harvested and fixed in 4% paraformaldehyde. After paraffin embedding, serial sections were prepared. Sections were dewaxed, rehydrated, and stained with hematoxylin for 3 min. A 1% hydrochloric acid ethanol solution was used for differentiation, followed by rinsing in distilled water. Eosin staining was performed for 5 min. Sections were dehydrated with absolute ethanol (twice), cleared, and sealed with neutral gum. After drying, scanned images were obtained using the 3DHISTECH Pannoramic MIDI pathological section scanner (3DHISTECH Ltd, Budapest, Hungary).

### 5.15. TUNEL Staining

After dewaxing and hydration, the paraffin sections were repaired with protease K for 30 min, and then the cell membrane was broken, and TUNEL dye was incubated for 1 h, followed by DAB for 10 min, hematoxylin staining for 2 min, dehydrated and sealed, and finally observed under a microscope.

### 5.16. Immunohistochemistry

The paraffin sections of the knee joint were dewaxed by xylene and gradient ethanol, then the antigenic repair was performed with citric acid repair solution, followed by BSA closure. Overnight incubation at 4 °C with the Conweishi immunohistochemical kit (CWBIO, Taizhou, China) and the primary antibody was used, then incubation for 2 h with the secondary antibody, color development was performed with DBA, and finally nuclear staining was performed with hematoxylin for 2 min, and the tablets were sealed after dehydration and transparency.

### 5.17. Immunofluorescence

After dewaxing the paraffin sections of the knee joint with xylene and gradient ethanol, the membrane was broken with 0.1% Triton reagent, the antigen was repaired with tissue fluorescence repair solution, and then the knee joint was sealed with BSA. Fluorescence dilution solution was used to dilute CD206 and CD86. After overnight incubation, fluorescent secondary antibody was used for incubation, and anti-fluorescent quenching sealing tablets containing DAPI were used to seal the tablets and filmed at the corresponding wavelength.

### 5.18. Enzyme-Linked Immunosorbent Assay (ELISA)

After drug administration, blood was collected from the mice via orbital puncture. Samples were left at room temperature for 20 min, then centrifuged at 3000 rpm for 10 min. The supernatant was collected for analysis. ELISA kits targeting TNF-α and IL-6 were used for detection according to the manufacturer’s instructions.

### 5.19. Transwell Experiment

Matrigel was diluted to a concentration of 50 mg/L at a 1:8 ratio and applied to the bottom of the upper Transwell chamber. Once gel formation was complete, chambers were placed in a cell incubator at 37 °C for 3 h. A 10 g/L BSA solution was then added and incubated for 30 min to hydrate the gel. The full-length mouse HSPA8 coding sequence was cloned into the pcDNA3.1 vector, and the recombinant plasmid (pcDNA3.1-HSPA8, GenePharma, Suzhou, China) was transiently transfected into FLs using Lipofectamine 3000 (Invitrogen, USA) according to the manufacturer’s instructions. Cells transfected with the empty pcDNA3.1 vector served as the negative control. After 24 h, transfected cells were harvested for subsequent assays. Cells were then digested with trypsin and washed twice with PBS. Each group of FLs was seeded into the upper chamber at a density of 1 × 10^5^ cells/well. Complete medium containing 10% serum was added to the lower chamber. After 24 h of incubation, non-migrated cells were removed, and chambers were washed twice with PBS. Cells were fixed with absolute ethanol for 15 min at room temperature, then stained with 1 g/L crystal violet for 10 min. Excess stain was removed by PBS washing. The number of stained cells was counted in 10 randomly selected fields under an inverted microscope, and the average was calculated.

### 5.20. Scratch Experiment

FLs were seeded into six-well plates at a density of 5 × 10^5^ cells/well. After 12 h, cells adhered uniformly. A scratch was made across the monolayer using a sterile pipette tip and a ruler. Floating cells and debris were removed by washing with PBS. Images were captured at 0 h, 24 h, and 48 h to assess the degree of cell migration.

### 5.21. Mito Sox Red Cell Fluorescence Staining

MitoSOX Red dye (Thermo Scientific, Waltham, MA, USA) was diluted to 500 nM/mL with HBSS, and MitoTracker Green was diluted to 100 nM. Hoechst dye was diluted to 40 nM/mL. The mixed dye solution was added to the cells, which were incubated at 37 °C in 5% CO_2_ for 30 min. After staining, cells were washed twice with HBSS and observed under a fluorescence microscope.

### 5.22. JC-1 Dyeing

Cells were first washed with PBS and then incubated with 1 mL of culture medium and 1 mL of JC-1 dye solution, mixed thoroughly. After 20 min of incubation at 37 °C, the supernatant was discarded. Cells were then washed twice with JC-1 staining buffer and observed under a fluorescence microscope.

### 5.23. Modified Saffron-O and Fast Green Stain

Knee joint sections were routinely dewaxed and rehydrated. Freshly prepared Weigert’s iron hematoxylin solution was added for staining for 5 min, followed by a water rinse. Sections were differentiated in acid alcohol for 15 s and washed with distilled water for 10 min. Samples were then immersed in Fast Green solution for 5 min. Residual green stain was removed by a quick rinse with a weak acid solution for 10–15 s. Sections were subsequently immersed in Saffron-O solution for 5 min. After gradient dehydration, sections were cleared in xylene, sealed with optical resin, and observed under a light microscope.

### 5.24. EdU Cell Staining

After dilution of the EdU proliferation kit with culture medium at a 1:1 ratio, cells were incubated for 3 h. A total of 0.1 mL of fixing solution (PBS containing 3.7% formaldehyde) was added to each well and incubated at room temperature for 15 min. Fixative was removed, and wells were washed with 0.1 mL of BSA wash solution for 5 min, repeated three times. After washing, 0.1 mL of permeabilization solution (PBS containing 0.5% Triton X-100) was added and incubated for 15 min at room temperature. The solution was removed, and cells were washed again with 0.1 mL BSA wash solution, repeated twice. Finally, stained cells were observed under a fluorescence microscope.

### 5.25. Statistical Analysis

All experiments were performed in triplicate or more (*n* ≥ 3 for in vitro experiments and n = 6 for animal experiments) to ensure reproducibility, and data are presented as mean ± standard deviation (SD). Two-tailed t test, two-factor analysis of variance, logrank test, Spearman correlation test, or Fisher’s exact test were used for statistical analysis. *p* < 0.05 was considered statistically significant. Statistical analysis and mapping were performed using GraphPad Prism 9.5.0 software.

## Data Availability

Data available on request from the authors: the data that support the findings of this study are available from the corresponding author upon reasonable request. Some data may not be made available because of privacy or ethical restrictions.

## Supplementary Materials

The following supporting information can be downloaded at: https://www.mdpi.com/article/10.3390/ijms27072946/s1.

## Abbreviations

AKT, Akt kinase; ASE, Artemisia sieversiana Ehrh; BMD, Bone Mineral Density; BP, Biological Process; BV, bone volume; CC, Cellular Component; CCK-8, Cell Counting Kit-8; CD86, Cluster of Differentiation 86; CD206, Cluster of Differentiation 206; DS, Discovery Studio; ELISA, enzyme linked immunosorbent assay; ESS, Ephedra sinica Stapf; FLs, Fibroblast-like synoviocytes; GO, Gene Ontology; HSPA8, heat shock protein family A member 8; HE, Hematoxylin-eosin; IL-6, Interleukin-6; IL-1β, Interleukin 1 beta; JAR, Juniperus angosturana R.P. Adams; KEGG, Kyoto Encyclopedia of Genes and Genomes; MF, Molecular Function; MTX, Methotrexate; Micro-CT, Micro-Computed Tomography; MMP, Mitochondrial membrane potential; MPM, Myricaria platyphylla Maxim; Mrc-1, Mannose receptor C-type 1; PI3K, Phosphatidylinositol 3-kinase; PI, Propidium Iodide; RA, Rheumatoid Arthritis; RAM, Rhododendron anthopogonoides Maxim; Tb.N, trabecular number; Tb.Th, trabecular thickness; TNF-α, tumor necrosis factor α; TV, tissue volume; WGL, WuweiGanLu.

## References

[1] Smolen J.S., Aletaha D., McInnes I.B. (2016). Rheumatoid arthritis. Lancet.

[2] Diaz-Gonzalez F., Hernandez-Hernandez M.V. (2023). Artritis reumatoideRheumatoid arthritis. Med. Clin..

[3] Deng Y., Zheng H., Li B., Huang F., Qiu Y., Yang Y., Sheng W., Peng C., Tian X., Wang W. (2024). Nanomedicines targeting activated immune cells and effector cells for rheumatoid arthritis treatment. J. Control. Release.

[4] Tascilar K., Hagen M., Kleyer A., Simon D., Reiser M., Hueber A.J., Manger B., Englbrecht M., Finzel S., Tony H.P. (2021). Treatment tapering and stopping in patients with rheumatoid arthritis in stable remission (RETRO): A multicentre, randomised, controlled, open-label, phase 3 trial. Lancet Rheumatol..

[5] Nygaard G., Firestein G.S. (2020). Restoring synovial homeostasis in rheumatoid arthritis by targeting fibroblast-like synoviocytes. Nat. Rev. Rheumatol..

[6] Alivernini S., MacDonald L., Elmesmari A., Finlay S., Tolusso B., Gigante M.R., Petricca L., Di Mario C., Bui L., Perniola S. (2020). Distinct synovial tissue macrophage subsets regulate inflammation and remission in rheumatoid arthritis. Nat. Med..

[7] Hua P., Liang R., Yang S., Tu Y., Chen M. (2024). Microneedle-assisted dual delivery of PUMA gene and celastrol for synergistic therapy of rheumatoid arthritis through restoring synovial homeostasis. Bioact. Mater..

[8] The L.R.H.P. (2024). Implementing pathogen genomics in the Western Pacific region: Evidence is needed. Lancet Reg. Health–West. Pac..

[9] Li S., Fang H.X., Wan L., Liu J. (2025). Role of Non-coding RNAs in Rheumatoid Arthritis and Supervision Mechanism of Chinese Medicine. Chin. J. Integr. Med..

[10] Gu Z.X., Wang Z.H., Zhang X.Q., Zhou G.H., Liang G.H., Zeng L.F., Liu J. (2025). Research advances in the study of traditional Chinese medicine formula granules on signaling pathway-mediated disease mechanisms. Front. Pharmacol..

[11] Wu N., Yuan T., Yin Z., Yuan X., Sun J., Wu Z., Zhang Q., Redshaw C., Yang S., Dai X. (2022). Network Pharmacology and Molecular Docking Study of the Chinese Miao Medicine Sidaxue in the Treatment of Rheumatoid Arthritis. Drug Des. Dev. Ther..

[12] Ba X., Huang Y., Shen P., Huang Y., Wang H., Han L., Lin W.J., Yan H.J., Xu L.J., Qin K. (2021). WTD Attenuating Rheumatoid Arthritis via Suppressing Angiogenesis and Modulating the PI3K/AKT/mTOR/HIF-1alpha Pathway. Front. Pharmacol..

[13] Wang T., Yang J., Chen X., Zhao K., Wang J., Zhang Y., Zhao J., Ga Y. (2017). Systems Study on the Antirheumatic Mechanism of Tibetan Medicated-Bath Therapy Using Wuwei-Ganlu-Yaoyu-Keli. BioMed Res. Int..

[14] Wen Y., Zhang S., Meng X., Zhao C., Hou B., Zhu X., Cai W., Zhou Y., Qiu L., Sun H. (2024). Water extracts of Tibetan medicine Wuweiganlu attenuates experimental arthritis via inducing macrophage polarization towards the M2 type. J. Ethnopharmacol..

[15] Zhang S., Hou B., Xu A., Wen Y., Zhu X., Cai W., Han Z., Chen J., Nhamdriel T., Mi M. (2024). Ganlu formula ethyl acetate extract (GLEE) blocked the development of experimental arthritis by inhibiting NLRP3 activation and reducing M1 type macrophage polarization. J. Ethnopharmacol..

[16] Hassani S.S., Farhadi E., Esmaeili S.A., Mahmoudi M., Mahmoudi M., Afshari J.T., Akhlaghi M., Jamshidi A. (2025). Deciphering the role of ERK and PI3K/Akt as crosstalk pathways between fibroblast-like synoviocytes and osteoclasts; novel therapeutic approach for rheumatoid arthritis. Mol. Biol. Rep..

[17] Yue S., Fan J., Xie D., Cao C., Wang Z., Huang J., Qiu F., Yang X., He D., Lu A. (2025). Unveiling the Therapeutic Potential: Targeting Fibroblast-like Synoviocytes in Rheumatoid Arthritis. Expert Rev. Mol. Med..

[18] Linghu K., Cui W., Li T., Tuo Y., Wang D., Pan H., Zhang T., Lin L., Yu H., Hu X. (2024). Small molecule α-methylene-γ-butyrolactone, an evolutionarily conserved moiety in sesquiterpene lactones, ameliorates arthritic phenotype via interference DNA binding activity of NF-κB. Acta Pharm. Sin. B.

[19] Meyer A., Zack S.R., Nijim W., Burgos A., Patel V., Zanotti B., Volin M.V., Amin M.A., Lewis M.J., Pitzalis C. (2024). Metabolic reprogramming by Syntenin-1 directs RA FLS and endothelial cell-mediated inflammation and angiogenesis. Cell. Mol. Immunol..

[20] Qin Y., Cai M.L., Jin H.Z., Huang W., Zhu C., Bozec A., Huang J., Chen Z. (2022). Age-associated B cells contribute to the pathogenesis of rheumatoid arthritis by inducing activation of fibroblast-like synoviocytes via TNF-alpha-mediated ERK1/2 and JAK-STAT1 pathways. Ann. Rheum. Dis..

[21] Chang B.Y., Jung Y.S., Yoon C.S., Oh J.S., Hong J.H., Kim Y.C., Kim S.Y. (2017). Fraxin Prevents Chemically Induced Hepatotoxicity by Reducing Oxidative Stress. Molecules.

[22] Li W., Li W., Yu J., Liu F., Zang L., Xiao X., Zhao J., Yao Q., Niu X. (2020). Fraxin inhibits lipopolysaccharide-induced inflammatory cytokines and protects against endotoxic shock in mice. Fundam. Clin. Pharmacol..

[23] Li W., Li W., Zang L., Liu F., Yao Q., Zhao J., Zhi W., Niu X. (2019). Fraxin ameliorates lipopolysaccharide-induced acute lung injury in mice by inhibiting the NF-kappaB and NLRP3 signalling pathways. Int. Immunopharmacol..

[24] Tang F.L., Xie L.W., Tang L.F., Lu H.Y., Zhu R.Q., Wang D.F., Tian Y., Cai S., Li M. (2024). Fraxin (7-hydroxy-6-methoxycoumarin 8-glucoside) confers protection against ionizing radiation-induced intestinal epithelial injury in vitro and in vivo. Int. Immunopharmacol..

[25] Liu Y., Pan Y.F., Xue Y.Q., Fang L.K., Guo X.H., Guo X., Liu M., Mo B.Y., Yang M.R., Liu F. (2018). uPAR promotes tumor-like biologic behaviors of fibroblast-like synoviocytes through PI3K/Akt signaling pathway in patients with rheumatoid arthritis. Cell. Mol. Immunol..

[26] Lin Y., Tang Y., Yi O., Zhu J., Su Z., Li G., Zhou H., Liu L., Liu B., Cai X. (2024). Graphene oxide quantum dots-loaded sinomenine hydrochloride nanocomplexes for effective treatment of rheumatoid arthritis via inducing macrophage repolarization and arresting abnormal proliferation of fibroblast-like synoviocytes. J. Nanobiotechnol..

[27] Li N., Li X., Deng L., Yang H., Gong Z., Wang Q., Pan D., Zeng S., Chen J. (2023). 6-Shogaol inhibits the proliferation, apoptosis, and migration of rheumatoid arthritis fibroblast-like synoviocytes via the PI3K/AKT/NF-kappaB pathway. Phytomedicine.

[28] Meng Q., Du X., Wang H., Gu H., Zhan J., Zhou Z. (2017). Astragalus polysaccharides inhibits cell growth and pro-inflammatory response in IL-1beta-stimulated fibroblast-like synoviocytes by enhancement of autophagy via PI3K/AKT/mTOR inhibition. Apoptosis.

[29] Wang L., He C. (2022). Nrf2-mediated anti-inflammatory polarization of macrophages as therapeutic targets for osteoarthritis. Front. Immunol..

[30] Wang C.B., Wong C.K., Ip W.K., Li M.L., Tian Y.P., Lam C.W. (2005). Induction of IL-6 in co-culture of bronchial epithelial cells and eosinophils is regulated by p38 MAPK and NF-kappaB. Allergy.

[31] Chiu L.C., Wang J.Y., Lin C.H., Hsu C.H., Lin L.C., Fu S.L. (2021). Diterpenoid Compounds Isolated from Chloranthus oldhamii Solms Exert Anti-Inflammatory Effects by Inhibiting the IKK/NF-kappaB Pathway. Molecules.

[32] Ross E.A., Devitt A., Johnson J.R. (2021). Macrophages: The Good, the Bad, and the Gluttony. Front. Immunol..

[33] Ferdous J., Bhuia M.S., Chowdhury R., Rakib A.I., Aktar M.A., Al H.M., Melo C.H., Islam M.T. (2024). Pharmacological Activities of Plant-Derived Fraxin with Molecular Mechanisms: A Comprehensive Review. Chem. Biodivers..

[34] Azietaku J.T., Ma H., Yu X.A., Li J., Oppong M.B., Cao J., An M., Chang Y.X. (2017). A review of the ethnopharmacology, phytochemistry and pharmacology of Notopterygium incisum. J. Ethnopharmacol..

[35] Xia X., Zeng H., Wang H., Li X., Zhang S., Yang L., He J. (2023). Revealing the Active Constituents and Mechanisms of Semiliquidambar cathayensis Chang Roots against Rheumatoid Arthritis through Network Pharmacology, Molecular Docking, and in Vivo Experiment. Chem. Biodivers..

[36] Li W., Wang K., Liu Y., Wu H., He Y., Li C., Wang Q., Su X., Yan S., Su W. (2022). A Novel Drug Combination of Mangiferin and Cinnamic Acid Alleviates Rheumatoid Arthritis by Inhibiting TLR4/NFκB/NLRP3 Activation-Induced Pyroptosis. Front. Immunol..

[37] Xin P., Xu X., Zhang H., Hu Y., Deng C., Sun S., Liu S., Zhou X., Ma H., Li X. (2023). Mechanism investigation of Duhuo Jisheng pill against rheumatoid arthritis based on a strategy for the integration of network pharmacology, molecular docking and in vivo experimental verification. Pharm. Biol..

[38] Li J.M., Zhang X., Wang X., Xie Y.C., Kong L.D. (2011). Protective effects of cortex fraxini coumarines against oxonate-induced hyperuricemia and renal dysfunction in mice. Eur. J. Pharmacol..

[39] Chen R., Zeng J., Li C., Xiao H., Li S., Lin Z., Huang K., Shen J., Huang H. (2022). Fraxin Promotes the Activation of Nrf2/ARE Pathway via Increasing the Expression of Connexin43 to Ameliorate Diabetic Renal Fibrosis. Front. Pharmacol..

[40] Qian Z., Ru X., Liu C., Huang X., Sun Q. (2021). Fraxin Prevents Knee Osteoarthritis through Inhibiting Chondrocyte Apoptosis in an Experimental Rat Osteoarthritis Model. Protein Pept. Lett..

[41] Beucher L., Gabillard-Lefort C., Baris O.R., Mialet-Perez J. (2024). Monoamine oxidases: A missing link between mitochondria and inflammation in chronic diseases?. Redox Biol..

[42] Venegas F.C., Sanchez-Rodriguez R., Luisetto R., Angioni R., Viola A., Canton M. (2024). Oxidative Stress by the Mitochondrial Monoamine Oxidase B Mediates Calcium Pyrophosphate Crystal-Induced Arthritis. Arthritis Rheumatol..

[43] Guo W.Y., Wu Q.M., Zeng H.F., Chen Y.L., Xu J., Yu Z.Y., Shu Y.K., Yang X.N., Zhang C.H., He X.Z. (2025). A sinomenine derivative alleviates bone destruction in collagen-induced arthritis mice by suppressing mitochondrial dysfunction and oxidative stress via the NRF2/HO-1/NQO1 signaling pathway. Pharmacol. Res..

[44] Lee S.Y., Moon J., Lee A.R., Moon Y.M., Choi J.W., Lee C.R., Jeon S.B., Sohn H.S., Youn J., Shin D. (2025). mtSTAT3 suppresses rheumatoid arthritis by regulating Th17 and synovial fibroblast inflammatory cell death with IL-17-mediated autophagy dysfunction. Exp. Mol. Med..

[45] Kim E.K., Kwon J.E., Lee S.Y., Lee E.J., Kim D.S., Moon S.J., Lee J., Kwok S.K., Park S.H., Cho M.L. (2017). IL-17-mediated mitochondrial dysfunction impairs apoptosis in rheumatoid arthritis synovial fibroblasts through activation of autophagy. Cell Death Dis..

[46] Lin J., He Y., Wang B., Xun Z., Chen S., Zeng Z., Ou Q. (2019). Blocking of YY1 reduce neutrophil infiltration by inhibiting IL-8 production via the PI3K-Akt-mTOR signaling pathway in rheumatoid arthritis. Clin. Exp. Immunol..

[47] Cheng W., Hao C., Zhao S., Zhang L., Liu D. (2019). SNHG16 promotes the progression of osteoarthritis through activating microRNA-93-5p/CCND1 axis. Eur. Rev. Med. Pharmacol. Sci..

[48] Cheung T.T., McInnes I.B. (2017). Future therapeutic targets in rheumatoid arthritis?. Semin. Immunopathol..

[49] Guo C., He J., Song X., Tan L., Wang M., Jiang P., Li Y., Cao Z., Peng C. (2019). Pharmacological properties and derivatives of shikonin-A review in recent years. Pharmacol. Res..

[50] Liu C., He L., Wang J., Wang Q., Sun C., Li Y., Jia K., Wang J., Xu T., Ming R. (2020). Anti-angiogenic effect of Shikonin in rheumatoid arthritis by downregulating PI3K/AKT and MAPKs signaling pathways. J. Ethnopharmacol..

[51] Chen K., Lin Z.W., He S.M., Wang C.Q., Yang J.C., Lu Y., Xie X.B., Li Q. (2019). Metformin inhibits the proliferation of rheumatoid arthritis fibroblast-like synoviocytes through IGF-IR/PI3K/AKT/m-TOR pathway. Biomed. Pharmacother..

[52] Liang Y., Li H., Gong X., Ding C. (2020). Long Non-coding RNA THRIL Mediates Cell Growth and Inflammatory Response of Fibroblast-Like Synoviocytes by Activating PI3K/AKT Signals in Rheumatoid Arthritis. Inflammation.

[53] Lin W., Liu Y., Zhang S., Xu S., Qiu Q., Wang C., Liu D., Shen C., Xu M., Shi M. (2023). Schisandrin treatment suppresses the proliferation, migration, invasion, and inflammatory responses of fibroblast-like synoviocytes from rheumatoid arthritis patients and attenuates synovial inflammation and joint destruction in CIA mice. Int. Immunopharmacol..

[54] Lin L., Huang Z., Li W., Liu X., Li X., Gao S., Chen J., Yang C., Min X., Yang H. (2024). Mid1 promotes synovitis in rheumatoid arthritis via ubiquitin-dependent post-translational modification. Pharmacol. Res..

[55] Yang J., Li S., Li Z., Yao L., Liu M., Tong K.L., Xu Q., Yu B., Peng R., Gui T. (2024). Targeting YAP1-regulated Glycolysis in Fibroblast-Like Synoviocytes Impairs Macrophage Infiltration to Ameliorate Diabetic Osteoarthritis Progression. Adv. Sci..

[56] Li Z., Zeng H., Jin B., Zhang M., Zhang X., Chai Y. (2025). Trends in rheumatoid arthritis burden in China and globally, 1990-2021: A longitudinal study based on the GBD database. PLoS ONE.

[57] Wu Y., Li X., Jin Y., Mao F., Zhang L. (2026). Exosomal non-coding RNAs in autoimmune diseases: Molecular mechanisms and potential applications. Front. Immunol..

[58] Mo W., Zhang Y., Zeng Y., Zheng Y., Zhao S., Wang X., Fan X. (2026). Extracellular vesicles in autoimmune diseases: Mechanistic underpinnings and precision medicine applications. Autoimmun. Rev..

[59] Buttari B., Recalchi S., Riitano G., Capozzi A., Ucci F.M., Manganelli V., Fratini F., Profumo E., Garofalo T., Alessandri C. (2025). Extracellular microvesicles from patients with Rheumatoid arthritis promote dendritic cell activation in vitro. Front. Immunol..

[60] Zhu X.X., Xu A.J., Cai W.W., Han Z.J., Zhang S.J., Hou B., Wen Y.Y., Cao X.Y., Li H.D., Du Y.Q. (2025). NaHS@Cy5@MS@SP nanoparticles improve rheumatoid arthritis by inactivating the Hedgehog signaling pathway through sustained and targeted release of H_2_S into the synovium. J. Nanobiotechnol..

[61] Feng J.H., Li L., Lv X.Y., Xiong F., Hu X.L., Wang H. (2022). Protective Effects of 4-Trifluoromethyl-(E)-cinnamoyl]-L-4-F-phenylalanine Acid against Chronic Cerebral Hypoperfusion Injury through Promoting Brain-Derived Neurotrophic Factor-Mediated Neurogenesis. ACS Chem. Neurosci..

[62] Yin Y., Yang X., Wu S., Ding X., Zhu H., Long X., Wang Y., Zhai S., Chen Y., Che N. (2022). Jmjd1c demethylates STAT3 to restrain plasma cell differentiation and rheumatoid arthritis. Nat. Immunol..

