# Supplementary material for: Fraxin Attenuates Rheumatoid Arthritis by Regulating Macrophage Polarization and Inhibiting Fibroblast-like Synoviocyte Proliferation
Source: Int J Mol Sci. 2026 Mar 24;27(7):2946. doi: 10.3390/ijms27072946 (PMC13073824; doi:10.3390/ijms27072946)
Supplement: Supplementary file 1 [file ijms-27-02946-s001.zip › ijms-4196584-supplementary.pdf]

Supplementary data

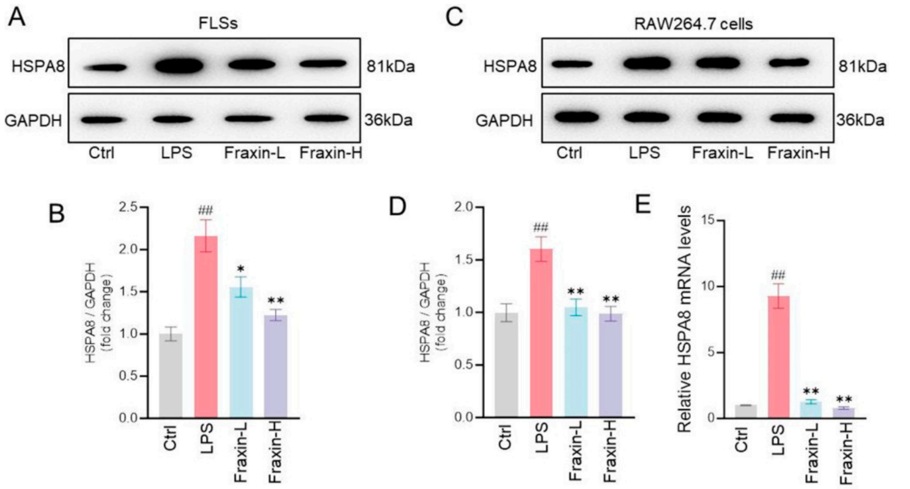

Figure S1. **Effects of Fraxin on the protein and mRNA expression levels of HSPA8**. (A-B) Detection of HSPA8 protein expression in FLs. (C-D) Detection of HSPA8 protein expression in RAW264.7 cells. (E) Detection of HSPA8 mRNA level in RAW264.7 cells. Fraxin low-dose group (Fraxin-L, 100ng/mL), Fraxin high-dose group (Fraxin-H, 1 μg/mL). ##P<0.01 versus Ctrl; * P<0.05 or ** P<0.01versus LPS.

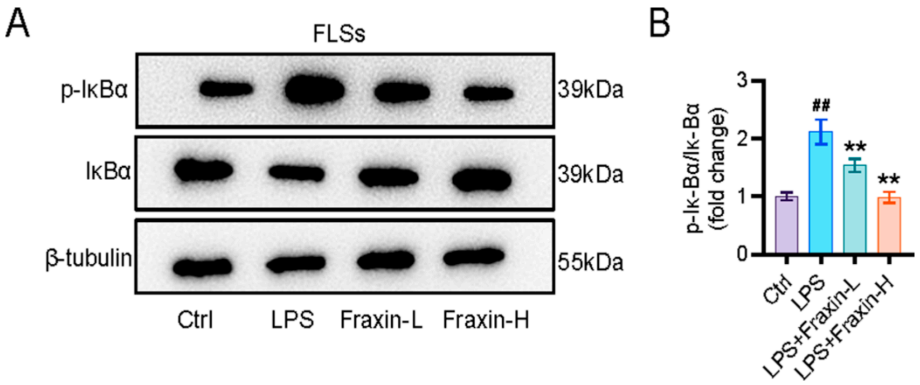

Figure S2. **Effects of Fraxin on the IκBα phosphorylation and degradation in FLs**. (A-B) Detection of phosphorylated and total protein expression of IκBα. Fraxin low-dose group (Fraxin-L, 100ng/mL), Fraxin high-dose group (Fraxin-H, 1 μg/mL). ##P<0.01 versus Ctrl; ** P<0.01versus LPS.

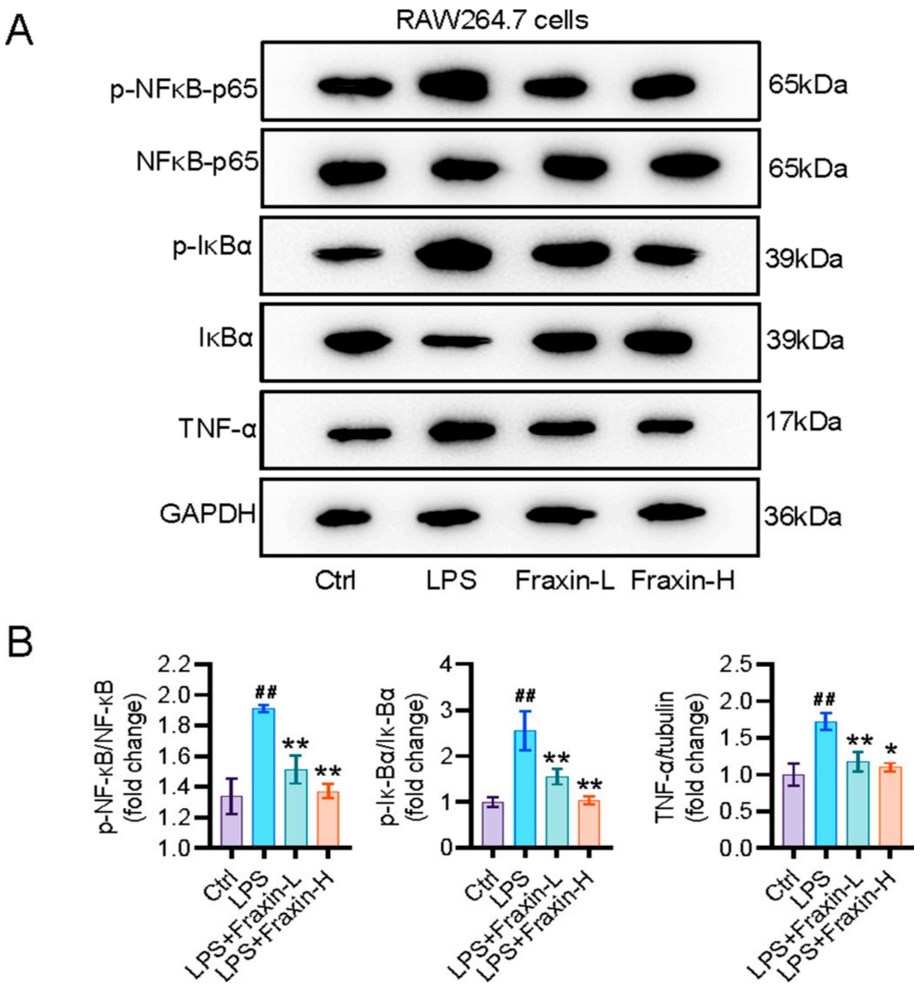

Figure S3. **Effects of Fraxin on the IκBα/NFκB signaling pathway in RAW264.7 cells**. (A-B) Detection of p-NFκB-p65, NFκB-p65, p-IκBα, IκBα, and TNF-α in LPS-exposed RAW264.7 cells. Fraxin low-dose group (Fraxin-L, 100ng/mL), Fraxin high-dose group (Fraxin-H, 1 μg/mL). ##P<0.01 versus Ctrl; ** P<0.01, * P<0.05 versus LPS.

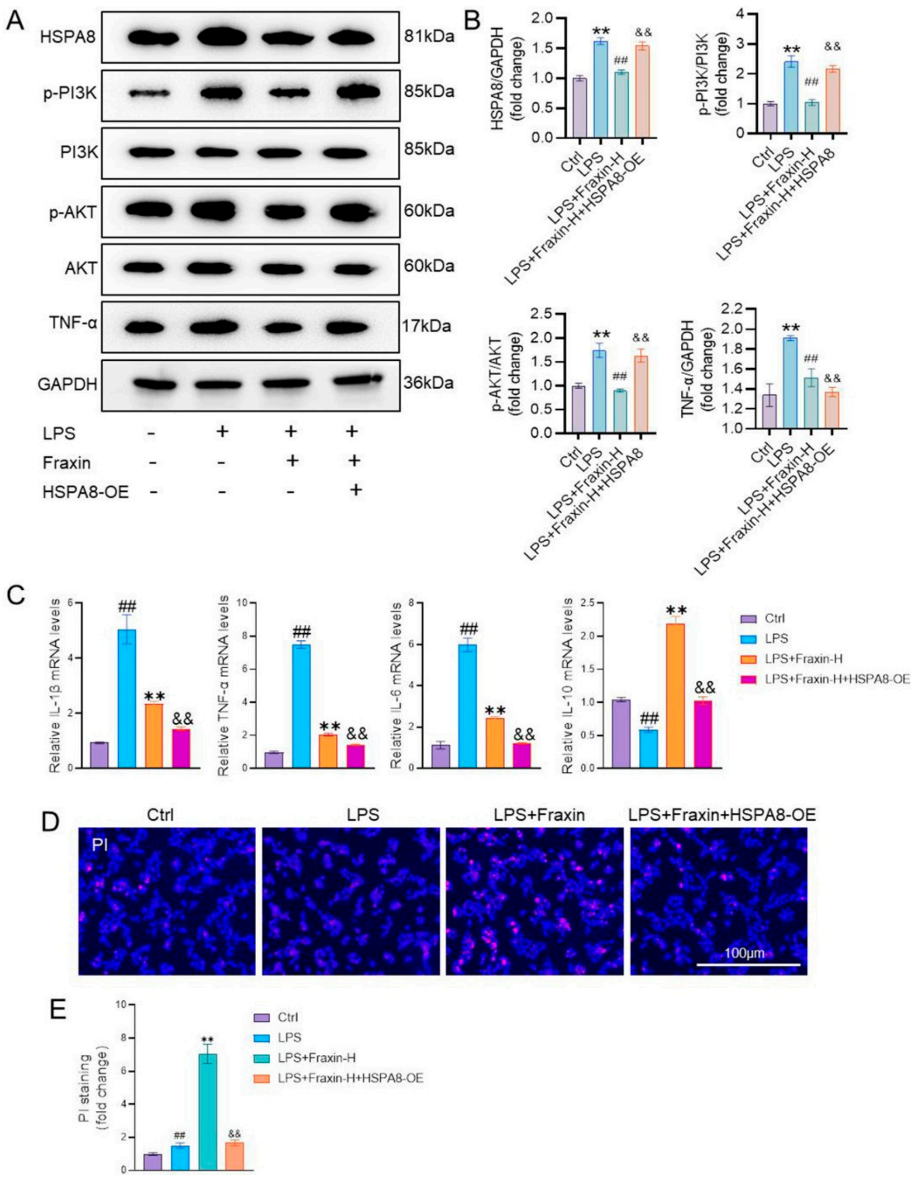

Figure S4. **Overexpression of HSPA8 reversed the effects of Fraxin on PI3K/AKT activation, inflammation, and apoptosis in FLs**. (A-B) Detection of HSPA8, p-PI3K, PI3K, p-AKT, AKT, and TNF-α protein expression in LPS-exposed FLs. (C) Detection of IL-1β, TNF-α, IL-6, and IL-10 mRNA levels in LPS-exposed FLs. (D-E) Detection of the apoptosis of FLs. Fraxin high-dose group (Fraxin-H, 1 μg/mL). ##P<0.01 versus Ctrl; ** P<0.01versus LPS; && P<0.01versus LPS+Fraxin-H.

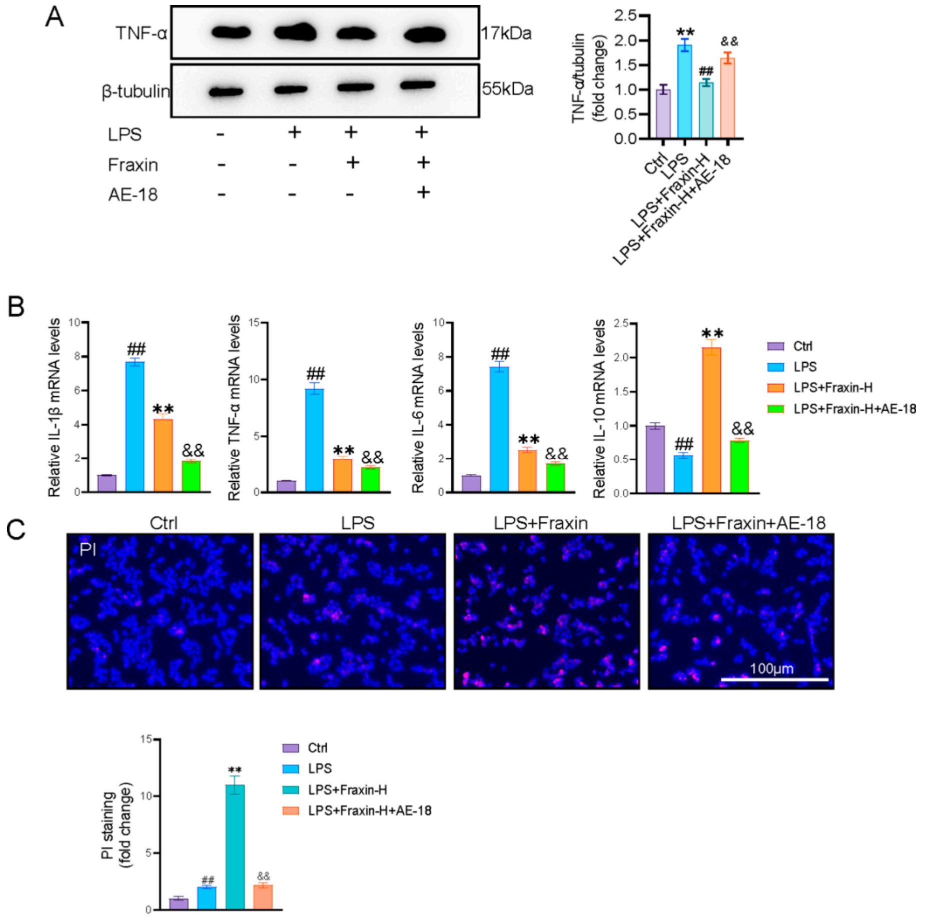

Figure S5. **The PI3K agonist AE-18 reversed the effects of Fraxin on inflammation and apoptosis in FLs**. (A) Detection of TNF-αprotein expression in LPS-exposed FLs. (B) Detection of *Il1b*, *Tnfa*, *Il6*, and *Il10* mRNA levels in LPS-exposed FLs. (C) Detection of the apoptosis of FLs. Fraxin high-dose group (Fraxin-H, 1 μg/mL). ##P<0.01 versus Ctrl; ** P<0.01versus LPS; && P<0.01versus LPS+Fraxin-H.
